# Phenotyping of Human Melanoma Cells Reveals a Unique Composition of Receptor Targets and a Subpopulation Co-Expressing ErbB4, EPO-R and NGF-R

**DOI:** 10.1371/journal.pone.0084417

**Published:** 2014-01-29

**Authors:** Irina Mirkina, Emir Hadzijusufovic, Clemens Krepler, Mario Mikula, Diana Mechtcheriakova, Sabine Strommer, Alexander Stella, Erika Jensen-Jarolim, Christoph Höller, Volker Wacheck, Hubert Pehamberger, Peter Valent

**Affiliations:** 1 Ludwig Boltzmann Cluster Oncology, Medical University of Vienna, Vienna, Austria; 2 Division of Hematology & Hemostaseology, Department of Medicine I, Medical University of Vienna, Vienna, Austria; 3 Department/Clinic for Companion Animals and Horses, Clinic for Small Animals, Clinical Unit of Internal Medicine, University of Veterinary Medicine Vienna, Austria; 4 Department of Dermatology, Medical University of Vienna, Vienna, Austria; 5 Institute of Medical Genetics, Medical University of Vienna, Vienna, Austria; 6 Department of Pathophysiology & Allergy Research, Medical University of Vienna, Vienna, Austria; 7 Department of Clinical Pharmacology, Medical University of Vienna, Vienna, Austria; 8 Comparative Medicine, Messerli Research Institute, University of Veterinary Medicine, Medical University of Vienna and University Vienna, Vienna, Austria; University of Tennessee, United States of America

## Abstract

Malignant melanoma is a life-threatening skin cancer increasingly diagnosed in the western world. In advanced disease the prognosis is grave. Growth and metastasis formation in melanomas are regulated by a network of cytokines, cytokine-receptors, and adhesion molecules. However, little is known about surface antigens and target expression profiles in human melanomas. We examined the cell surface antigen profile of human skin melanoma cells by multicolor flow cytometry, and compared their phenotype with 4 melanoma cell lines (A375, 607B, Mel-Juso, SK-Mel28). Melanoma cells were defined as CD45−/CD31− cells co-expressing one or more melanoma-related antigens (CD63, CD146, CD166). In most patients, melanoma cells exhibited ErbB3/Her3, CD44/Pgp-1, ICAM-1/CD54 and IGF-1-R/CD221, but did not express CD20, ErbB2/Her2, KIT/CD117, AC133/CD133 or MDR-1/CD243. Melanoma cell lines were found to display a similar phenotype. In most patients, a distinct subpopulation of melanoma cells (4–40%) expressed the erythropoietin receptor (EPO-R) and ErbB4 together with PD-1 and NGF-R/CD271. Both the EPO-R+ and EPO-R− subpopulations produced melanoma lesions in NOD/SCID IL-2Rgamma^null^ (NSG) mice in first and secondary recipients. Normal skin melanocytes did not express ErbB4 or EPO-R, but expressed a functional KIT receptor (CD117) as well as NGF-R, ErbB3/Her3, IGF-1-R and CD44. In conclusion, melanoma cells display a unique composition of surface target antigens and cytokine receptors. Malignant transformation of melanomas is accompanied by loss of KIT and acquisition of EPO-R and ErbB4, both of which are co-expressed with NGF-R and PD-1 in distinct subfractions of melanoma cells. However, expression of EPO-R/ErbB4/PD-1 is not indicative of a selective melanoma-initiating potential.

## Introduction

Malignant melanoma is a life-threatening skin cancer with rapidly increasing incidence in industrialized countries worldwide [1]–[4]. While early-stage melanomas can often be cured by surgery, the prognosis in advanced disease is still grave in all patients [5]–[7]. In particular, metastatic melanoma lesions are largely resistant against conventional chemotherapy, targeted drugs or/and radiation. Therefore, current research is attempting to reveal novel mechanisms underlying disease initiation and evolution as well as progression, metastasis-formation and drug resistance in human melanomas [8]–[10].

Growth, adhesion, and survival of melanoma cells supposedly are regulated by a network of cytokines, cytokine-receptors, and surface adhesion molecules [11]–[14]. Such receptors and their ligands may contribute substantially to growth and migration of melanoma cells as well as metastasis formation in advanced disease. However, so far, little is known about the distribution of cytokine receptors, adhesion molecules and other surface antigens on primary melanoma cells [11]–[14]. From studies performed with cell lines and cultured cells, several cytokine receptors, including ErbB family-members and the erythropoietin receptor (EPO-R) were reported to be expressed in melanomas [15]–[17]. However, most of these studies were performed on cultured and passaged melanoma cells or cell lines. More recently, several attempt have been made to better define cytokine receptor profiles for primary patient-derived melanoma cells and melanoma-initiating cells [18]–[20].

Long-term melanoma growth and metastasis-formation supposedly depend on the presence of melanoma-initiating cells that have unlimited capacity for self-renewal and proliferation [18]–[20]. These melanoma-initiating cells were considered to reside within distinct subpopulations. In fact, first reports suggested that melanoma-initiating cells represent a minority of all cells in a given melanoma lesion [18]–[20]. However, Quintana et al described that approximately 30–40% of all melanoma cells have melanoma-initiating potential in NOD/SCID IL-2Rgamma^null^ (NSG) mice [21]. Based on this information, we and others have screened for melanoma markers that are expressed in a substantial subset of melanoma cells and exhibit a particular or even a selective melanoma-initiating capacity. Recent data of Boiko et al. have suggested that melanoma-initiating cells express the nerve growth factor (NGF) receptor CD271 [22]. Other studies have suggested that CD271-negative cells can also form melanoma lesions in NSG mice, and that both the phenotype and stemness of melanoma cells may be reversible features [23], [24]. However, most of these studies have been performed with cell lines or with *in vitro*-transformed melanoma cells.

In the present study, we have examined the phenotype and cytokine receptor profile of freshly obtained, patient-derived human skin melanoma cells. Unexpectedly, the stem cell factor receptor KIT was only detectable on normal melanocytes but not on melanoma cells. We also found that in most patients, distinct subfractions of melanoma cells co-express EPO-R, CD24, and ErbB4, and that EPO-R expression overlaps with NGF-R expression. However, both the EPO-R-positive as well as the EPO-R-negative cell fractions were found to produce melanoma lesions in NSG mice in primary and secondary recipients. Together, our data suggest that malignant transformation in melanomas may be associated with loss of KIT and expression of EPO-R and ErbB4. However, none of these cytokine receptors seem to be indicative for a particular melanoma-initiating potential.

## Materials and Methods

### Monoclonal antibodies (mAb) and other reagents

A specification of mAb used in this study is shown in Table 1. The ErbB-targeting drugs canertinib, BIBW2992, and BMS-599626, the KIT kinase blockers nilotinib, midostaurin (PKC412) and dasatinib, and the insulin-like growth factor-1 receptor (IGF-1-R) blocker OSI-906, were from Chemietek (Indianapolis, IN). The irreversible ErbB inhibitor pelitinib and the CD33-targeting antibody-conjugate gemtuzumab ozogamicin (mylotarg) were kindly provided by Wyeth (Cambridge, MA). Recombinant human (rh) stem cell factor (SCF) was purchased from Peprotech (Rocky Hill, NJ), rh erythropoietin (EPO) and rh transforming growth factor-β1 (TGF-β1) from R&D Systems (Minneapolis, MN), and rh Heregulin-α (EGF-domain) from Sigma-Aldrich (St. Louis, MO). Dulbecco's modified eagle medium (DMEM) and fetal calf serum (FCS) were from Gibco Life Technologies (Gaithersburg, MD).

**Table 1:** Specification of monoclonal antibodies (mAb).

Clone	CD	Antigen	Fluoro-chrome	Ig class	Source	Reactivity	Company
38409	-	EPO-R	PE	IgG2b	mouse	human	R&D
EGFR.1	-	EGF-R1 (ErbB1/HER1)	PE	IgG2b	mouse	human	BD
191924	-	EGF-R2 (ErbB2/HER2)	PE	IgG2b	mouse	human	R&D
66223	-	EGF-R3 (ErbB3/HER3)	PE	IgG1	mouse	human	R&D
182818	-	EGF-R4 (ErbB4/HER4)	PE	IgG2a	mouse	human	R&D
182818		EGF-R4 (ErbB4/HER4)	APC	IgG2a	mouse	human	R&D
95106	-	c-MET (HGF-R)	PE	IgG1	mouse	human	R&D
W6/32	-	HLA-A,B,C	APC	IgG2a	mouse	human	BioLegend
L27	20	CD20	PE	IgG1	mouse	human	BD
ML5	24	CD24	FITC	IgG2a	mouse	human	BD
AC128	31	PECAM-1	APC	IgG1	mouse	human	Miltenyi Biotec
390	31	PECAM-1	FITC	IgG2a	rat	mouse	BD
WM53	33	Siglec-3	PE	IgG1	mouse	human	BD
581	34	HPCA-1	PE	IgG1	mouse	human	BD
515	44	Hyaluronan-R, Pgp-1	PE	IgG1	mouse	human	BD
2D1	45	LCA	PerCP	IgG1	mouse	human	BD
30-F11	45	LCA	PerCP	IgG2b	rat	mouse	BD
HA58	54	ICAM-1	PE	IgG1	mouse	human	BD
CLB-grant12	63	LAMP-3	PE	IgG1	mouse	human	Immunotech
283340	66a	CEACAM-1	PE	IgG2b	mouse	human	R&D
166707	105	Endoglin	PE	IgG1	mouse	human	R&D
LMM741	114	G-CSF-R	PE	IgG1	mouse	human	BD
61708	115	CSF-1-R/M-CSF-R	PE	IgG1	mouse	human	R&D
31916	116	GM-CSF-RA	PE	IgG1	mouse	human	R&D
104D2	117	KIT, SCF-R	PE	IgG1	mouse	human	BD
32703	123	IL-3-RA	PE	IgG1	mouse	human	R&D
AC133	133	Prominin-1, AC133	PE	IgG1	mouse	human	Miltenyi Biotec
BV10A4H2	135	FLT-3	PE	IgG1	mouse	human	Biolegend
128018	146	Mel-CAM (MCAM, MUC18)	PE	IgG1	mouse	human	R&D
128018	146	Mel-CAM (MCAM, MUC18)	FITC	IgG1	mouse	human	R&D
105902	166	ALCAM	PE	IgG1	mouse	human	R&D
eBio5G3	171	L1 Antigen/NCAM-1	PE	IgG2a	mouse	human	eBioscience
33255	221	IGF-I-R	PE	IgG1	mouse	human	R&D
15D3	243	MDR1/P-glycoprotein	PE	IgG1	mouse	human	BD
C40-1457	271	NGF-R/P75	PE	IgG1	mouse	human	BD
EH12.2H7	279	PD-1	PE	IgG1	mouse	human	BioLegend
89106	309	VEGF-R2/KDR	PE	IgG1	mouse	human	R&D
5D3	338	ABCG2	PE	IgG2b	mouse	human	BioLegend

Abbreviations: PE, phycoerythrin; FITC, fluorescein isothiocyanate; PerCP, peridinin chlorophyll protein; APC, allophycocyanin; EPO-R, erythropoietin receptor; EGF-R, epidermal growth factor receptor; HGF-R, hepatocyte growth factor receptor; IGF-I-R, insulin-like growth factor I receptor; PD-1, programmed death-1; NGF-R, nerve growth factor receptor; HLA, human leukocyte antigens; PECAM-1, platelet/endothelial cell adhesion molecule-1; Siglec-3, sialic acid binding immunoglobulin-like lectin-3; HPCA-1, human progenitor cell antigen-1; LCA, leukocyte common antigen; ICAM-1, intercellular adhesion molecule-1; LAMP-3, lysosomal-associated membrane protein 3; CEACAM-1, carcinoembryonic antigen-related cell adhesion molecule 1; G-CSF-R, granulocyte colony-stimulating factor receptor; CSF-1-R, colony stimulating factor 1 receptor; GM-CSF-RA, granulocyte/macrophage colony-stimulating factor receptor alpha chain; SCF, stem cell factor; IL-3RA, Interleukin 3 receptor alpha chain; FLT-3, FMS-like tyrosine kinase 3; Mel-CAM (MCAM), melanoma cell adhesion molecule; ALCAM, activated leukocyte cell adhesion molecule; L1 Antigen/NCAM-1, leukocyte cell adhesion antigen-1/neural cell adhesion molecule-1); MDR-1, multidrug resistance protein-1; VEGF-R2/KDR, vascular endothelial growth factor receptor-2/kinase insert domain receptor; ABCG2, ATP-binding cassette (ABC) transporter subfamily G member 2; BD, Becton Dickinson.

### Melanoma cells and melanoma cell lines

The following cell types were analyzed: a) freshly isolated skin melanoma cells (9 patients with metastatic and 1 with primary melanoma), b) cultured patient-derived melanoma cells (2^nd^ to 4^th^ passage), c) 4 human melanoma cell lines (A375, Mel-Juso, SK-Mel28, 607B), and d) patient-derived melanoma cells grown in NOD/SCID/IL-2γ^null^ (NSG) mice. The cell lines Mel-Juso and SK-Mel28 are originating from the ATCC (LGC Standards GmbH, Wesel, Germany), the cell line A375 from DSMZ (Braunschweig, Germany) and the cell line 607B was kindly provided by Dr.Schrier; University of Leiden, Leiden, Netherlands (original reference: Jansen et al.; PNAS, 1999). All patients gave written informed consent before surgery. The patients' characteristics are shown in Table S1a in File S1. The study was approved by the local ethics committee of the Medical University of Vienna. Skin samples were cut into small pieces and washed in Tyrode's buffer. Then, samples were incubated in collagenase type 2 (Worthington, Lakewood, NJ) (1.5 mg/ml) at 37°C for 3 hours. After incubation, FCS (10%) was added, and dispersed cells centrifuged (1,400 rpm, 10 minutes), washed, resuspended in DMEM plus 10% FCS, and passed through a cell-strainer (40 µM). After isolation, cells were examined for viability (usually >90%), and were either cultured or examined for cell surface marker expression. Normal foreskin-derived melanocytes (3 caucasian donors) were purchased from CellMade (Archamps, France) or PromoCell (Heidelberg, Germany) and cultured in serum-free Melanocyte Growth Medium (MGM, CellMade). Cells were detached and passaged by mild trypsinization with Subculture Reagent Kit, according to the recommendations of the manufacturer (CellMade). Patient-derived melanoma cells were cultured in DMEM and 10% FCS in collagen-coated flasks. Melanoma cell lines were cultured in DMEM containing 10% FCS and L-glutamine. Cells were passaged using 0.05% trypsin-EDTA (Gibco). All cells were cultured in 5% CO_2_ at 37°C.

### Multicolor flow cytometry and cell sorting

Patient-derived melanoma cells and xenotransplanted melanoma cells were analyzed by multicolor flow cytometry using various combinations of fluorochrome-conjugated mAb (1 µL per 10^5^ cells) shown in Table S1b in File S1. Melanoma cells were defined as CD45−/CD31− cells co-expressing at least one of the following melanoma markers: CD63, CD146, or CD166. The flow-strategy used to define melanoma cells in skin samples is shown in Figure S1. Melanoma cell lines were analyzed by single-color flow cytometry. Human serum IgG (Sigma, St. Louis, MO) was used to block Fc receptors. Cells were incubated with mAb at 4°C for 30 minutes, washed and then examined on a FACSCalibur™ (BD Biosciences). Staining-reactions were controlled using isotype-matched antibodies and analyzed by Flow Jo software (Tree Star, Ashland, OR). Results were expressed as mean fluorescence intensity (MFI) and as percentage of positive cells. To confirm EPO-R expression, melanoma cells were labeled with biotinylated rh EPO according to the recommendations of the manufacturer (R&D Systems). In a separate set of experiments, EPO-R-positive and EPO-R-negative fractions of melanoma cells (3 patients) were purified by cell sorting on a FACSAria (BD Biosciences). The purity of sorted EPO-R+ (positive) cells amounted to >99.5%, and in the EPO-R-negative sort, less than 1% of all melanoma cells exhibited the EPO-R.

### Melanoma-initiation in NOD/SCID/IL-2Rγ−/− (NSG) mice

In order to define the melanoma-initiating potential of subpopulations of primary patient-derived melanoma cells, highly purified EPO-R-positive and EPO-R-negative fractions of cells were injected into the skin of NSG mice (Jackson Laboratory, Bar Harbor, ME) as described by Quintana et al [24]. Animal studies were approved by the ethics committee of the Medical University of Vienna, and carried out in accordance with guidelines for animal care and protection and protocols approved by Austrian law (GZ 680 205/67-BrGt/2003). About 1–5×10^5^ melanoma cells (each mouse) were injected subcutaneously (in 100 µl matrigel, BD Biosciences) into the right flanks of 5-week-old NSG mice (4–6 mice per group). Mice were inspected for tumor formation for up to 6 months. Tumor size was measured with a size-calliper, and the tumor volume (V) calculated as V = W^2^×L×0.5 (W = width; L = length). Tumor-bearing mice were sacrificed, and the recovered melanoma cells examined for their surface antigen phenotype (markers listed in Table 1), response to cytokines, and response to targeted drugs. In select experiments, tumor-derived melanoma cells (from initially EPO-R-positive and initially EPO-R-negative fractions in patient #6) were injected into secondary recipient NSG mice under the (same) conditions described above.

### Real-time PCR

Melanoma cells (5×10^5^) were lyzed in 1.0 mL Trizol® (Invitrogen, Paisley, UK). Total RNA was isolated according to the manufacturer's protocol (Life Technologies, Paisley, UK) utilizing Phase Lock Gel™ extraction tubes (Eppendorf AG, Hamburg, Germany). cDNA was prepared using High Capacity cDNA Reverse Transcription kit (Applied Biosystems, Warrington, UK) according to the protocol of the manufacturer. Real-time PCR was performed on ABI PRISM 7900HT using SYBR Green detection system (Applied Biosystems, Warrington, UK) essentially as described [25]. Primers were designed using “Primer Express 2.0” software (Applied Biosystems). Primer sequences are depicted in Table S1c in File S1. Housekeeping genes (HKG) included eukaryotic translation elongation factor 1α1 (EF1A), actin beta (ACTB), and ribosomal protein (RPLP0). Each PCR reaction was performed in triplicates. For relative quantification, data were analyzed by ΔΔCT and ΔCT methods using SDS 2.1 software (Applied Biosystems). Expression levels of target genes were normalized to HKG expression levels (mean level of all 3 HKG examined).

### Incubation of melanoma cells with cytokine ligands and targeted drugs

Patient-derived melanoma cells (xenotransplanted or sorted cells), normal skin melanocytes, and melanoma cells lines (A375, Mel-Juso, SK-Mel28, 607B) were kept in serum-free medium with 0.5% bovine serum albumin (BSA) (Gibco) in 96-well microplates (1×10^4^ cells/well) overnight for growth factor-starvation. Cell lines were then incubated with rh EPO (5 U/ml), TGF-β1 (100 ng/ml), Heregulin-α (100 ng/ml), SCF (100 ng/ml), or control medium for 48 hours. Melanocytes (1.8×10^4^ cells per well) were exposed to EPO (5 U/ml) or SCF (100 ng/ml) with or without nilotinib (1 µM) for 48 hours. In a separate set of experiments, primary, sorted EPO-R-positive and EPO-R-negative melanoma cells were incubated with 50 ng/ml of NGF, 10 U/ml EPO, or a combination of NGF and EPO (same concentrations) at 37°C for 48 hours. The following targeted drugs were applied on cell lines or patient-derived melanoma cells: canertinib, pelitinib, MCS2156119J-15, BIBW2992, BMS-599626, nilotinib, PKC412, dasatinib, gemtuzumab-ozogamicin, and OSI-906 (each 0.1–10 µM). Cells were incubated with or without drugs in complete medium plus 10% FCS at 5% CO_2_ (37°C) for 48 or 72 hours. All experiments were performed in triplicates.

### Evaluation of proliferation and survival of melanoma cells

After incubation with cytokines or/and drugs, 0.5 µCi ^3^H-thymidine (Amersham, Buckinghamshire, UK) were added for 16 hours. Cells were then harvested on filter membranes (Packard Bioscience, Meriden, CT) in a Filtermate 196 harvester (Packard Bioscience). Filters were air-dried and bound radioactivity counted in a β-counter (Top-Count NXT, Packard Bioscience). ^3^H-thymidine uptake was expressed as percent of medium control. Cell survival was assessed by Vybrant MTT proliferation kit (Molecular Probes, Eugene, OR). In brief, cells were seeded in 96-well-microplates (1×10^4^ cells per well) in 180 µl DMEM plus 10% FCS. After incubation with cytokines or drugs for 48 or 72 hours, cells were discarded, and 100 µl phenol red-free IMDM added to each well, followed by addition of 10 µl MTT solution (12 mM) for 4 hours. After incubation, 10% SDS in 0.01 M HCl (100 µL) was added to each well (overnight). Optical density (OD) was measured at 570 nm in a Spectramax microplate reader (Molecular Devices) using SoftMax Pro version 5 software. Results were expressed as percent viable cells relative to cells kept in control medium.

### Evaluation of apoptosis by flow cytometry and TUNEL assay

Primary melanoma cells and cell lines (A375, SK-Mel28, Mel-Juso, 607B) were incubated in control medium or in medium containing various concentrations of targeted drugs (see above) for 48 hours (TUNEL assay) or 72 hours (Annexin V-FITC). After incubation, cells were detached by trypsinization. For flow cytometric evaluation of apoptosis, cells were stained with Annexin V-FITC and analyzed on a FACSCalibur™. Results were expressed in percentage of AnnexinV-positive (apoptotic) cells. For TUNEL assay, 7×10^4^ cells were spun on cytospin slides (Star Frost, Laborchemie GmbH). The TUNEL assay was performed using *in situ* cell death fluorescein detection kit (Roche, Mannheim, Germany) following the manufacturer's protocol. Briefly, slides were fixed with 4% paraformaldehyde (60 minutes), washed, and permeabilized with permeabilization buffer (0.1% Triton X-100 in 0.1% sodium citrate) at 4°C for 5 minutes. Then, slides were washed and incubated with 30 µl TUNEL reagent at 37°C for 60 minutes. After washing, nuclei were counter-stained with TO-PRO-3 (100 nM) (Invitrogen) for 5 minutes. Then, slides were washed and mounted using Vectashield mounting medium (Vector Laboratories). Slides were analyzed by a fluorescent laser scanning (LSM510) confocal microscope Axiovert 200M (Carl Zeiss, Jena, Germany) using AIM version 4.2 software (Carl Zeiss).

### Statistical analysis

To determine the significance in differences in growth and percentage of apoptotic cells the student's t test for dependent samples was applied. Results were considered statistically significant when p was <0.05.

## Results

### Phenotypic definition of melanoma cells in primary tumor samples

Patient-derived melanoma cells were defined as CD45−/CD31− cells co-expressing CD146 and/or CD166 and/or CD63. In most cases, all three markers were found to be expressed on melanoma cells, and there were no major differences in expression of ‘melanoma markers’ when comparing freshly isolated, passaged, or xenograft-derived cells. Melanoma cell lines were found to stain uniformly positive for CD146 and CD166. The percentage of CD63+ cells in melanoma cell lines was 39% in 607B cells, 42% in A375 cells, 87% in SK-Mel28 cells, and 89% in Mel-Juso cells. Normal skin melanocytes were also found to express CD146 (>95% cells), CD166 (>95% cells) and CD63 (30–40% cells reactive).

### Expression of cytokine receptors on melanoma cells

**Figure 1:**
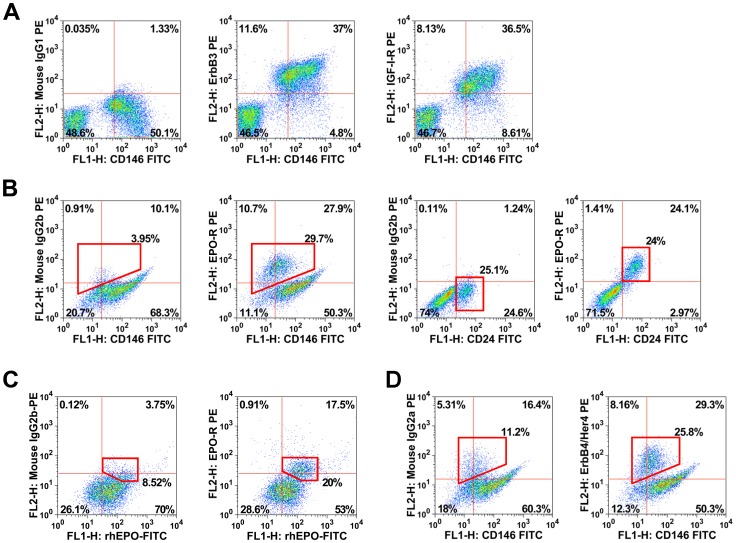
Primary melanoma cells express ErbB3 and ErbB4 as well as the EPO-R. *A*, Freshly isolated primary melanoma cells (patient #3) were stained with fluorochrome-conjugated monoclonal antibodies (mAb) against CD31, CD45, ErbB3, IGF-1-R, and CD146. Melanoma cells were gated as CD31−/CD45− cells and defined as CD146+ cells. Dot plots show expression of ErbB3 (middle panel) and IGF-1-R (right panel) on CD146+ melanoma cells. The isotype control is also shown (left panel). *B*, Melanoma cells of patient #10 were stained with mAb against CD31, CD45 and CD146 as well as EPO-R and CD24. The dot plot in the right panel shows co-expression of EPO-R and CD24 in a distinct subpopulation of (CD146+) melanoma cells. *C*, Xenotransplanted EPO-R+ melanoma cells of patient #6 were stained with biotinylated recombinant human EPO, an isotype-matched mouse IgG2b-PE antibody (left panel) and a mAb directed against the EPO-R (right panel). *D*, Melanoma cells of patient #10 were stained with mAb against CD146, an isotype-matched control antibody (left panel) and an antibody against ErbB4 (right panel). Dot plots show expression of ErbB4 in a distinct subpopulation of melanoma cells.

**Figure 2:**
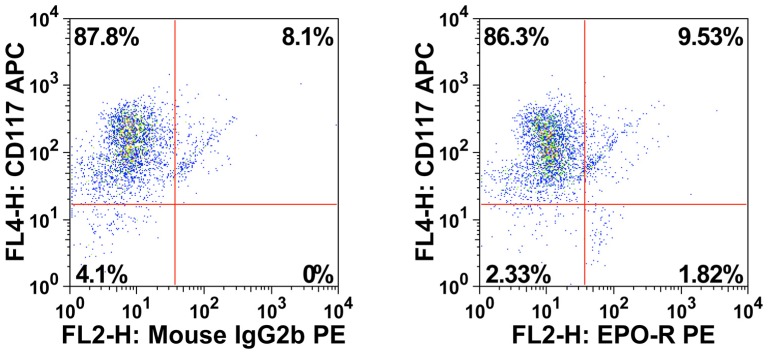
Expression of KIT/CD117 on human melanocytes. Normal epidermal foreskin melanocytes were stained with fluorochrome-conjugated monoclonal antibodies (mAb) directed against CD31, CD45 and CD146 for melanocyte detection, and mAb against KIT and EPO-R. Specificity of the staining reaction was controlled by applying an isotype-matched IgG2b antibody (left panel). As visible, melanocytes expressed KIT but did not express substantial amounts of EPO-R.

A number of cytokine receptors were found to be expressed on human melanoma cells. The ErbB3 receptor was expressed on freshly isolated melanoma cells in all samples tested, and in most samples of *in vitro* cultured or xenograft-derived melanoma cells (Table 2, Table S1d in File S1 and Figure 1A). The IGF-1-R was expressed on melanoma cells in 6 of 15 patient-derived melanoma samples tested, namely in 3 samples of freshly isolated cells and in 3 xenograft-samples (Table 2, Table S1d in File S1 and Figure 1A). The EPO-R was found to be expressed on freshly isolated melanoma cells in all patients examined (Table 2, Table S1d in File S1, Figure 1B). However, the levels of EPO-R on melanoma cells varied from patient to patient (range: 4% to 40%) (Table S1d in File S1). In contrast to other antigens, the EPO-R was found to be expressed on a distinct subpopulation of melanoma cells co-expressing CD24 (Figure 1B). Expression of the EPO-R on melanoma cells could also be confirmed by using directly labeled (biotinylated) rh EPO (Figure 1C). Another cytokine receptor detectable in a distinct subpopulation of melanoma cells was ErbB4 (Table 2, Table S1d in File S1, Figure 1D). Endoglin (CD105), a component of the TGFß1-R complex, was expressed on cultured and xenotransplant-derived melanoma cells in all patients (range: 32–63%), whereas in freshly isolated melanoma cells, CD105 was only expressed at very low or undetectable levels (Table S1d in File S1). None of the other cytokine receptors tested were consistently expressed on primary melanoma cells (Table 2 and Table S1d in File S1). Melanoma cell lines were found to display a similar profile of cytokine receptors compared to primary cells (Table 2 and Table S1d in File S1). By contrast, normal melanocytes differed markedly in cytokine receptor expression. In particular, normal melanocytes expressed high levels of SCF-R KIT (CD117), but did not express EPO-R or ErbB4 (Table 2, Table S1d in File S1 and Figure 2). All in all, melanoma cells display a unique composition of cytokine receptors and differ from normal melanocytes in expression of EPO-R, ErbB4, and KIT.

**Table 2:** Expression of cytokine- and growth factor receptors on melanoma cells.

	VEGF-R2	ErbB1	ErbB2	ErbB3	ErbB4	IGFI-R	Endoglin	KIT	EPO-R	c-MET	G-CSF-R	GM-CSF-R	M-CSF-R	IL-3-RA	FLT3
**MELANOMA CELL LINES**															
A375	−	−	−	++	−	++	++	−	−	++	−	−	−	−	−
607B	−	−	−	++	−	+	++	−	−	++	−	−	−	−	−
Mel-Juso	−	−	−	++	−	++	++	−	−	+	−	−	−	−	−
SK-Mel28	−	−	−	++	−	+	++	−	−	+/−	−	−	−	−	−
**PATIENT-DERIVED MELANOMA CELLS**															
freshly isolated	−	−	−	+	+/−	+	−/+	−	+/−	−	−	n.t.	n.t.	n.t.	n.t.
	n^a^ = 6	n = 7	n = 6	n = 7	n = 3	n = 6	n = 7	n = 9	n = 8	n = 2	n = 1				
xenografted^b^	−	−	+/−	++	+/−	+/−	+/−	+	+	−	n.t.	−	−	−	−
	n = 2	n = 2	n = 2	n = 2	n = 2	n = 2	n = 2	n = 2	n = 2	n = 2		n = 2	n = 2	n = 2	n = 2
*in vitro* cultured	n.t.	−	−	+/−	n.t.	−	+	−	+/−	n.t.	n.t.	n.t.	n.t.	n.t.	n.t.
		n = 6	n = 6	n = 9		n = 9	n = 6	n = 9	n = 5						
**NORMAL HUMAN MELANOCYTES**															
	n.t.	n.t.	n.t.	++	−	+/−	++	++	−	n.t.	n.t.	n.t.	n.t.	n.t.	n.t.
				n = 2	n = 2	n = 1	n = 1	n = 5	n = 3						

Score of reactivity of melanoma cells with antibodies: −,<5% positive cells; −/+, weakly expressed on a subset; +/−, 6–20%; +, 21–60%, and ++, 61–100% of cells positive.

a Number of donors (n) analyzed.

b Cells were obtained from tumors grown in NSG mice injected with unfractionated patient-derived melanoma cells.

Abbreviations: VEGF-R2, vascular endothelial growth factor receptor-2; EPO-R, erythropoietin receptor; IGF-I-R, insulin-like growth factor I receptor; G-CSF-R, granulocyte colony-stimulating factor receptor; GM-CSF-R, granulocyte/macrophage colony-stimulating factor receptor; M-CSF-R, macrophage colony-stimulating factor receptor; SCF, stem cell factor; IL-3-RA, interleukin-3 receptor alpha chain; FLT-3, FMS-like tyrosine kinase 3; n.t., not tested.

### Expression of adhesion-related antigens on melanoma cells

The intercellular adhesion molecule-1 (ICAM-1) (CD54) was found to be expressed on primary melanoma cells in all patients (n = 6) examined (Table 3, Table S1e in File S1). Another important adhesion molecule, CEACAM-1 (CD66a) [21], was found to be expressed on melanoma cells in 5 out of 6 patients examined (Table S1e in File S1). By contrast, the adhesion receptor LA (CD171) was not detectable on patient-derived melanoma cells (Table 3, Table S1e in File S1). Similar to melanoma cells, all melanoma cell lines expressed high levels of ICAM-1 (CD54). By contrast, CEACAM-1 (CD66a) was only detected in 2 out of the 4 melanoma cell lines tested (Table 3, Table S1e in File S1).

**Table 3:** Expression of adhesion-related molecules on melanoma cells.

	ICAM-1/CD54	CEACAM-1/CD66a	L1/CD171
**MELANOMA CELL LINES**			
A375	++	−	++
607B	++	++	++
Mel-Juso	++	−	++
SK-Mel28	+	++	++
**PATIENT-DERIVED MELANOMA CELLS**			
freshly isolated	++	+	−
	n^a^ = 6	n = 6	n = 6
xenografted^b^	++	+	−
	n = 2	n = 2	n = 2

Score of reactivity of melanoma cells with antibodies: −, <5%; +/−, 6–20%; +, 21–60%, and ++, 61–100%.

a Number of donors (n) analysed.

b Cells were obtained from tumours grown in NSG mice injected with impure (non-sorted) patient-derived melanoma cells.

Abbreviations: ICAM-1, intercellular adhesion molecule-1; CEACAM-1, carcinoembryonic antigen-related cell adhesion molecule 1.

### Cytokine receptor mRNA expression levels in melanoma cells

To confirm expression of cytokine receptors and target antigens in melanoma cells at the mRNA level, quantitative polymerase chain reaction (qPCR) studies were performed on melanoma cell lines and xenograft-derived melanoma cells, using primers specific for various cytokine receptors and other marker antigens. In these analyses, we were able to confirm expression of transcripts specific for CD63, ErbB3, CD44, and IGF-1-R in melanoma cells and melanoma cell lines in all samples tested. As expected, EPO-R mRNA was found to be expressed in primary melanoma cells (not shown) whereas the melanoma cell lines examined expressed only low levels of EPO-R mRNA. All in all, mRNA expression data were found to correlate nicely with our immunostaining results. A summary of mRNA expression data obtained with melanoma cell lines is shown in Figure S2.

### Phenotypic heterogeneity: identification of a melanoma cell subpopulation that co-expresses the EPO-R and ErbB4 as well as NGF-R

**Figure 3:**
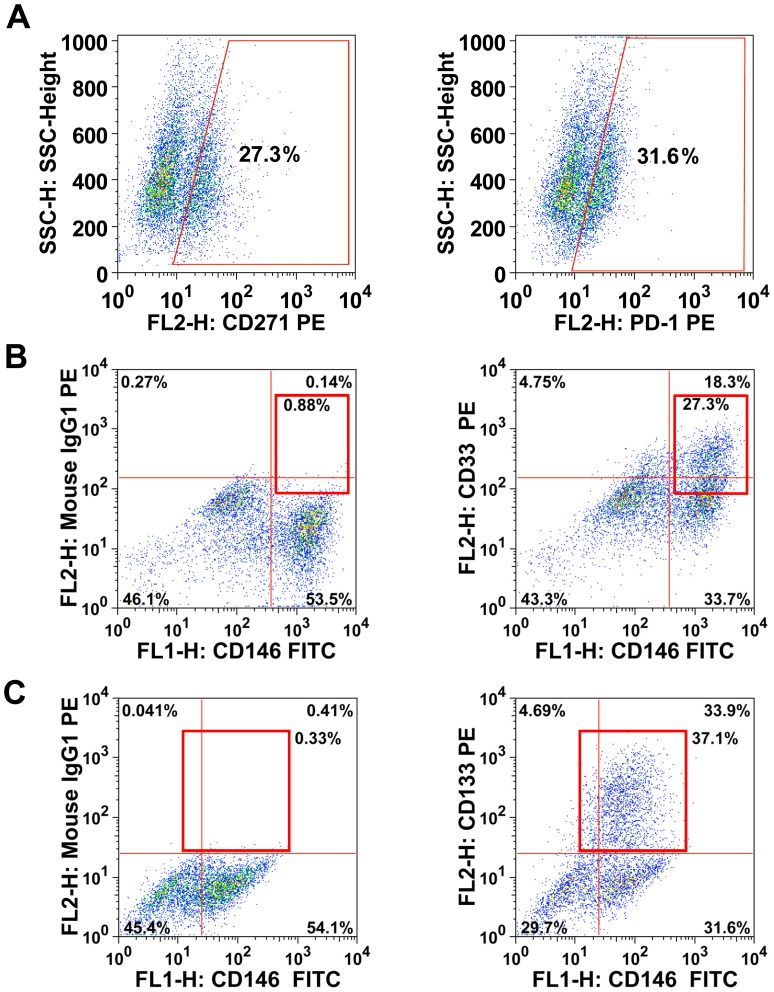
Expression of NGF-R, CD33 and CD133 on distinct populations of melanoma cells. *A*, Patient-derived melanoma cells (n = 3) were stained with a PE-labeled monoclonal antibody (mAb) against NGF-R (CD271) (left image) and a PE-labeled antibody against PD1 (right panel). Isotype-matched PE-labeled antibodies were used for determination of the cut-off values. *B*, Xenotransplanted melanoma cells from patient #6 were stained with mAb against CD33 and CD146. The dot plot shows expression of CD33 on a distinct subpopulation of CD146+ melanoma cells. *C*, Melanoma cells from patient #9 were stained with mAb against CD133 and CD146. The dot plot shows expression of CD133 on CD146+ melanoma cells. The reactivity of melanoma cells with isotype-matched control antibodies is shown in the left images of Fig. 3B and 3C.

In an attempt to identify phenotypically distinct melanoma cell subsets, we screened for markers that are differentially expressed on melanoma cell populations. The most obvious discriminating marker was the EPO-R. In fact, in all primary melanoma cell samples examined, a distinctively EPO-R-positive and a clearly EPO-R-negative subpopulation of cells was detected. The percentage of EPO-R-positive cells ranged between 4% and 40%. Another marker that was found to be expressed differentially on melanoma cells was CD24. This antigen was detectable on melanoma cells in 5 out of 6 patient-derived melanoma samples analyzed (Table S1f in File S1). The percentage of CD24+ cells ranged from 4% to 48% (Table 4). Interestingly, in all samples analyzed, CD24 was expressed on a subpopulation of melanoma cells that also co-expressed the EPO-R (Figure 1B). Moreover, this subpopulation of melanoma cells co-expressed ErbB4. Finally, we were able to show that this EPO-R+/CD24+/ErbB4+ subpopulation of melanoma cells co-expresses the putative melanoma stem cell antigen PD1 as well as the NGF-R (CD271) (Figure 3A). Another cell surface marker found to be differentially expressed on melanoma cells was Siglec-3 (CD33). This ‘myeloid’ antigen was expressed on a small but distinct subpopulation of patient-derived (cultured or freshly isolated) melanoma cells (4/12 samples), and in 2 out of 4 xenotransplant-derived melanoma cell populations (Table 4, Table S1e in File S1, Figure 3B). In freshly obtained melanoma samples, the percentage of CD33+ cells ranged from 1% to 7%, and in cultured or xenotransplanted melanoma cells, it ranged between 7% and 31% (Supplementary Table S1f in File S1). However, there was no correlation between expression of CD33 and expression of the EPO-R, CD24, or NGF-R. Other markers reportedly expressed on (repopulating) subfractions of melanoma cells, like CD20 or MDR-1, were hardly or not detectable on melanoma cells (Table 4 and Table S1f in File S1). The ABCG2 transporter showed a slight expression (5%) in 2 out of 13 patient-derived melanoma samples examined (Table S1f in File S1). The AC133 antigen, described to be indicative of melanoma-initiating cells, was not detectable on melanoma cells in most samples studied. However, unexpectedly, in 1 patient, a distinct subpopulation (37%) of all melanoma cells was found to display CD133 (Table 4, Table S1f in File S1, Figure 3C). In a majority of all patients examined, melanoma cells stained positive for CD44 (Table 4, Table S1f in File S1).

**Table 4:** Expression of potential stem cell markers and drug targets on melanoma cells.

	CD133	CD20	MDR-1	ABCG2	CD44	CD24	CD33	CD271	PD-1
**MELANOMA CELL LINES**									
A375	−	−	−	−	++	+/−	+	+	−
607B	−	−	−	−	++	+/−	+	++	−
Mel-Juso	−	−	−	−	++	+/−	−	+/−	−
SK-Mel28	−	−	−	−	++	+/−	−	+	−
**PATIENT-DERIVED MELANOMA CELLS**									
freshly isolated	−	−	−	−	++	+	−	+	+
	n^a^ = 10	n = 6	n = 6	n = 8	n = 8	n = 6	n = 8	n = 1	n = 1
xenografted^b^	−	−	−	−	++	+/−	+/−	−	+/−
	n = 2	n = 2	n = 2	n = 2	n = 2	n = 2	n = 2	n = 2	n = 2
*in vitro* cultured	−	−	−	−	+	n.t.	+/−	n.t.	n.t.
	n = 9	n = 3	n = 1	n = 5	n = 5		n = 4		
**NORMAL HUMAN EPIDERMAL MELANOCYTES**									
	−	n.t.	n.t.	−	++	−	−	+	−
	n = 1			n = 1	n = 1	n = 1	n = 1	n = 2	n = 2

Score of reactivity of melanoma cells with antibodies: −, <5%; +/−, 6–20%; +, 21–60%, and ++, 61–100%.

a Number of donors (n) analysed.

b Cells were obtained from tumors grown in NSG mice injected with the non-sorted patient-derived melanoma cells.

Abbreviations: n.t., not tested.

### Repopulation of melanoma cells in NSG mice

**Figure 4:**
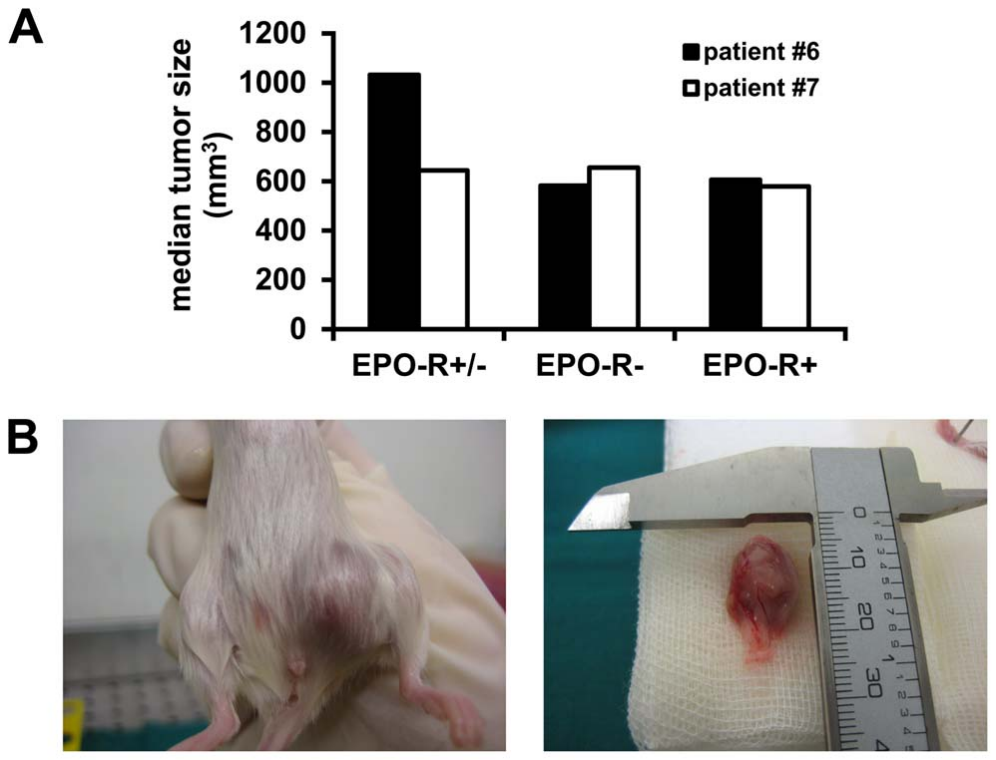
Melanoma-formation in NSG mice. Unsorted (EPO-R+/−) and sorted EPO-R− and EPO-R+, patient-derived, melanoma cells (patients #6 and #7) were injected subcutaneously into NSG mice (4–6 mice in each group). After 15 weeks, tumors were visible, mice were sacrificed, and the tumor-volumes were determined. *A*, Results show the median tumor-volumes (mm^3^) in each group. *B*, A melanoma cell-containing tumor formed in an NSG mouse by EPO-R− cells obtained from patient #6.

To test the hypothesis that a distinct subfraction of melanoma cells would have a particular capacity or lack the ability to form melanoma lesions in NSG mice, highly purified sorted cell fractions were injected into the skin of NSG mice. Since melanoma-repopulating (stem) cells, identified in NSG mice, have been described to represent about 25–30% of all melanoma cells in a given cell sample [22] we selected the EPO-R that was identified on up to 40% of all melanoma cells in our patient-derived samples. In two patients, melanoma cells were sorted as EPO-R-positive and EPO-R-negative subfractions, and injected into NSG mice. In both donors, the EPO-R-positive fraction was found to contain melanoma-initiating cells. In fact, EPO-R-positive cells formed melanoma lesions in NSG mice within 6–19 weeks, and these melanoma lesions were found to contain again melanoma-initiating cells when injected into secondary recipient mice. There were no differences in tumor size or weight when comparing tumor lesions produced by EPO-R+ cells and tumor lesions produced by EPO-R− cells, neither in the primary recipients (Figure 4) nor in the secondary recipients (not shown). Melanoma cells derived from the primary and secondary recipients were found to express the same phenotype when compared to the initial melanoma sample, including the EPO-R as well as ErbB4 and CD24, although expression of NGF-R was very low (Tables S1d and S1f in File S1). We then asked whether EPO-R-negative cells would also contain melanoma-initiating cells. Unexpectedly, melanoma-initiating cells were also detected in the EPO-R-negative fraction in both donors. Again, primary and secondary recipient mice were employed and were found to develop melanoma lesions after injection of EPO-R-negative cells. These data suggest that melanoma-initiating cells reside within both the EPO-R-positive and EPO-R-negative fractions of melanoma cells. An interesting observation was that some of the melanoma cells that engrafted from EPO-R-negative cells in NSG mice were found to express the EPO-R by flow cytometry (Table S1d in File S1).

### Effects of cytokines on expression of EPO-R on melanoma cells

We next asked whether cytokines would modulate expression of EPO-R in melanoma cells. For this purpose, melanoma cell lines were exposed to various cytokines (EPO, EGF, TGF-ß1, Heregulin-α, IGF-1, HGF, SCF, NGF) and then were subjected to qPCR analysis and flow cytometry. However, no effects of these cytokines on expression of EPO-R on melanoma cells were found. We also asked whether any of the cytokines would promote or modulate growth of human melanoma cells. However, we were unable to detect any significant effects of EPO, SCF, Heregulin-α or TGF-β1 on growth of melanoma cell lines (data not shown). However, recombinant SCF was found to promote growth of normal epidermal melanocytes, whereas recombinant EPO showed no effect on melanocyte growth (Figure S3). The growth-stimulating effect of SCF on melanocytes was specific in that proliferation was blocked by pre-treatment of cells with the KIT tyrosine kinase blocker nilotinib (Figure S3B).

### Effects of targeted drugs on melanoma cells

**Figure 5:**
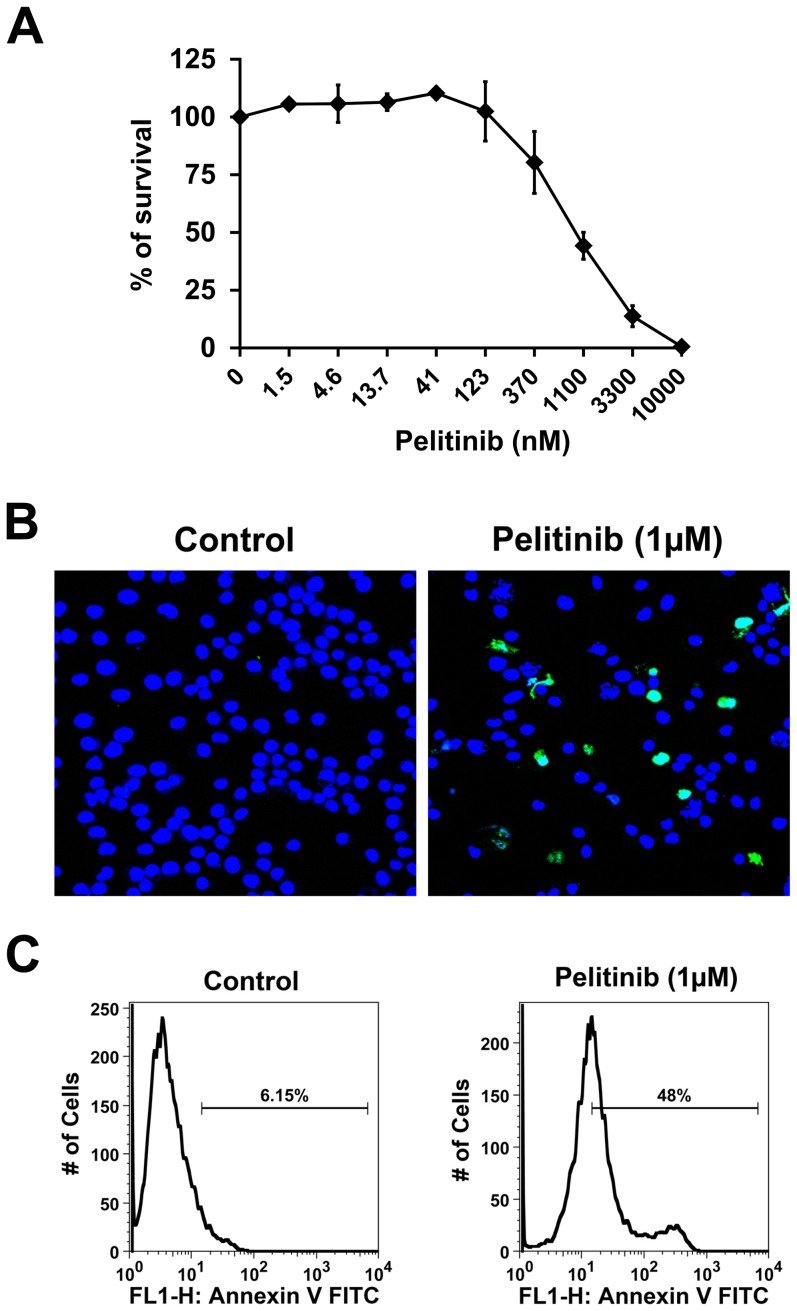
Pelitinib blocks survival and induces apoptosis in melanoma cells. *A*, A375 melanoma cells were incubated in control medium (0) or with increasing concentrations of the ErbB blocker pelitinib at 37°C for 72 hours. Cell survival was measured by MTT assay. Results are expressed as percent of control and represent the mean±S.D. of three independent experiments. *B*, Evaluation of apoptosis of melanoma cells by TUNEL assay. A375 cells were incubated in control medium (left panel) or in medium containing 1 µM pelitinib for 48 hours. As visible, the ErbB blocker induced apoptosis in A375 cells. Nuclei were counterstained by TO-PRO3 dye (blue color). *C*, Measurement of pelitinib-induced apoptosis in A375 cells by AnnexinV-staining. A375 cells were incubated in control medium (left panel) or medium containing pelitinib at 1 µM (right panel) for 72 hours. Histogram plots show the mean fluorescence intensity of the staining reaction. The percentages of apoptotic (AnnexinV-positive) cells are also provided.

To examine the potential role of identified drug targets on melanoma cells, we applied several drugs targeting these oncogenic receptors. Our screen included the irreversible pan-EGFR inhibitors canertinib, BIBW2992 and pelitinib, the reversible pan-EGFR inhibitor BMS-599626, the multikinase inhibitors dasatinib and PKC412, and the c-Met/HGF-R blocker MCS2156119J-15. First we evaluated the above listed targeted drugs in a MTT assay on melanoma cell lines. As shown in Table 5, the majority of the tested compounds only marginally reduced cell survival in melanoma cell lines. The most active compound was the EGFR blocker pelitinib that consistently suppressed cell survival in melanoma cell lines with an IC_50_ value in the low micromolar range (Table 5 and Figure 5A). We next examined pelitinib for its ability to induce apoptosis in melanoma cell lines. As exemplified in the Figure 5, incubation of the A375 melanoma cell line with 1 µM pelitinib resulted in substantial apoptosis evidenced by TUNEL assay (Figure 5B) and AnnexinV staining (Figure 5C).

**Table 5:** IC_50_ values (µM) for the inhibition of the cell survival (MTT assay) in human melanoma cells by targeted drugs.

	Dasatinib	PKC412	Pelitinib	BIBW2992	Canertinib	BMS-599626	OSI-906
**A375**	9.0–10.0	n.t.	0.8–1.0	2.0–2.5	4.0–5.0	no inhibition	>10
**SK-Mel28**	9.0–10.0	n.t.	2.0–3.2	3.0–5.0	7.0–8.0	7.0–8.0	no inhibition
**Mel-Juso**	>10	n.t.	0.9–1.2	2.0–2.5	5.0–7.0	>10.0	>10
**607B**	>10	n.t.	1.5–2.0	2.0–2.5	5.0–7.0	no inhibition	>10
**Patient #6** *	n.t.	>10	5	n.t.	n.t.	n.t.	no inhibition
**Patient #7** *	n.t.	>10	5	n.t.	n.t.	n.t.	no inhibition

Melanoma cells were cultured in complete medium at 37°C in the absence or presence of various concentrations of targeted drugs for 48 hours. Then, cell viability was examined by MTT assay as described in the text.

* xenotransplanted patient-derived melanoma cells from patient #6 and #7; n.t., not tested.

## Discussion

Various cell surface antigens have been implicated in the pathogenesis of human melanomas [11]–[13]. These molecules include cytokine receptors, adhesion molecules, ‘stem cell-associated’ antigens, and drug resistance-related antigens [11]–[13]. Most of these studies have employed melanoma cell lines. However, only a few studies have systematically analyzed expression of cell surface antigens on primary melanoma cells [14]–[17]. In the present study, we applied a larger number of mAb in order to examine the cell surface antigen phenotype of human melanomas. The results of our study show that melanoma cells display a unique composition of cell surface antigens, including ErbB3, IGF-1-R, CD44 and ICAM-1. In most donors, subpopulations of melanoma cells also expressed the EPO-R, CD24, and ErbB4. By contrast, in most patients, melanoma cells did not express the SCF-R KIT, G-CSF-R, IL-3-R or AC133. The only distinct and clearly separable subpopulation of melanoma cells (up to 40% of all cells) co-expressed EPO-R and CD24, and was found to contain melanoma-initiating cells when injected into NSG mice. However, the EPO-R-negative subpopulation was also found to contain melanoma-initiating cells, suggesting that EPO-R/CD24-expression is not indicative of a particular (specific) melanoma-initiating (melanoma stem cell) potential.

A number of recent studies suggest that melanoma cells may display a quite heterogeneous phenotype [20]–[24]. Therefore, it was important to establish a robust phenotypic definition for melanoma cells for flow cytometry studies. In the present study, melanoma cells were defined as CD45-negative and CD31-negative cells (excluding leukocytes and endothelial cells) that co-expressed at least one of the classical melanoma-related antigens, namely CD146, CD166 or CD63. Using this approach, even melanoma populations that expressed only low amounts of CD146 or CD166, or were largely negative for CD63, were included in our studies. However, on the other hand, we cannot exclude with certainty that in some of the patients examined, melanoma cell subpopulations (subclones) co-expressed CD31 or even CD45. On the other hand, most of the CD31+ or CD45+ cells usually did not co-express CD146 and CD166, which makes this possibility rather unlikely.

So far, little is known about phenotypic characteristics of malignant melanoma cells and phenotypic aberrancies occurring during melanoma evolution. In the current study, we compared the phenotype of primary melanoma cells with that of normal skin melanocytes. The results of our study show that normal melanocytes express huge amounts of KIT (CD117) on their surface, but do not express EPO-R, whereas in most patients, melanoma cells did not express detectable KIT, but expressed the EPO-R in a clearly detectable subpopulation. These data suggest a profound change in cytokine receptor expression during transformation of melanocytes into malignant melanomas. With regard to EPO-R expression, this observation confirms earlier studies in which spontaneous outgrowth of melanoma-like cell from melanocytes was accompanied by ‘*de novo*’ expression of the EPO-R [15]. Furthermore, recent data have shown that expression of EPO-R on melanoma cells is associated with enhanced growth and tumorigenicity of human melanomas [27]–[29].

The absence of KIT on primary human melanoma cells in most patients was a somehow unexpected finding. Notably, KIT-deficiency is associated with melanocyte deficiency. Moreover, normal skin melanocytes were stained by the same anti-KIT antibody and were found to grow better in medium containing the KIT ligand SCF. Finally, mucosal and acral melanomas have been described to express KIT and to harbor activating *KIT* mutations [30]. The failure to detect KIT on skin melanomas may have several explanations. First, KIT may induce differentiation rather than proliferation in melanocyte-committed progenitors, so that this pathway has to be bypassed or deleted by the melanoma-initiating oncogenic machinery. An alternative explanation may be that epigenetic mechanisms critical to melanoma-initiation automatically lead to silencing of the *KIT* gene. Ligand-blockage and selective cytoplasmic expression of KIT in melanoma cells could be excluded in the present study. In this regard it is also noteworthy that various KIT tyrosine kinase inhibitors failed to block the growth of melanoma cells. Therefore, whatever the mechanism of KIT-negativity in melanoma cells might be, KIT and KIT-dependent signaling apparently is neither critical to melanoma-initiation nor to progression or metastasis formation. In fact, neither the freshly isolated primary melanoma cells nor melanoma cells isolated from metastatic tumor cell lesions expressed KIT.

The expression of EPO-R on human melanoma cells has already been reported in several previous studies [15], [27]–[31]. In the present study, we confirmed EPO-R expression in melanoma cells by qPCR as well as surface staining experiments. In addition, we were able to show that the same melanoma cells that were recognized by the anti-EPO-R antibody also bind biotinylated recombinant EPO on their surface. These data provide solid evidence that primary melanoma cells express EPO-R. To define a functional role for the EPO-R on melanoma cells, we performed cell culture experiments using recombinant EPO. However, in the current study EPO was neither found to induce proliferation nor to enhance viability in melanoma cells.

A number of previous papers have suggested that melanoma-initiating cells display a unique phenotype, including CD133 [18], [20]–[23]. However, in most patients examined in our study, CD133 was neither expressed in a (small) subpopulation of melanoma cells nor in melanoma cell lines. More recent data suggest that up to 40% of, or even more, melanoma cells display melanoma-initiating capacity [22]. In the present study, two markers that were reproducibly detected in a distinct subpopulation of up to 40% of all melanoma cells were EPO-R and CD24. An interesting aspect was that these two antigens were found to be co-expressed in the same sub-populations of melanoma cells. In addition, these EPO-R+/CD24+ cells were found to co-express NGF-R CD271, a marker that has recently been reported as a potential marker of melanoma-initiating cells [23]. Therefore, we asked whether EPO-R+ melanoma cells are enriched in melanoma-initiating cells. To exclude contaminations, resorted cells exhibiting a purity of >99.9%, were injected into NSG mice in these experiments. However, melanomas were found to develop from both the EPO-R-positive and EPO-R-negative cells in NSG mice. An interesting finding was that the EPO-R was detectable not only in the xenotransplanted lesions derived from EPO-R+ cells, but also in melanoma cells derived from xenotransplanted EPO-R-negative fractions. This observation may be explained by the fact that melanoma cells may change their phenotype depending on microenvironmental factors such as hypoxia. Indeed, it has been described that melanoma (stem) cell phenotypes may change depending on various factor and the microenvironment [24]–[26], [31]. Alternatively, the EPO-R is required for long-term growth of melanomas and is expressed as soon as melanoma-initiating cells become activated and acquire stem cell function. The possibility that contaminating EPO-R+ cells were co-injected seems unlikely because of the high purity of injected cells. In a second step, we also performed experiments in secondary recipient mice. However, again, all mice injected were found to develop melanoma lesions, suggesting that EPO-R is not a specific marker or indicator of melanoma-propagating cells. These data are in line with more recent studies [22], [24], [25] and with the assumption that in advanced melanomas as well as in other advanced cancer types, the stem cell hierarchy is flat and many cells have or can acquire cancer-repopulating ability [31], [32].

Malignant melanoma is a clinical challenge, especially when metastases are found. In these patients, malignant cells are usually resistant against drug therapy and the prognosis is poor. Therefore, considerable effort is made to develop new drugs for patients with advanced melanomas [33]–[35]. In the present study, we asked whether melanoma cells express molecular targets and whether targeted drugs would induce growth arrest and apoptosis in melanoma cell lines. However, most of the target antigens were not expressed on melanoma cells. The few targets we were able to detect on melanoma cells were ErbB3, CD33 and CD44, confirming previous observations [20]–[23], [36]. However, growth-inhibitory effects were only seen with irreversible ErbB inhibitors, whereas no effects were seen with KIT blockers, IGF1-R-targeting drugs, or the CD33-antibody-toxin-conjugate gemtuzumab ocogamicin.

In conclusion, we have established the cell surface antigen phenotype of human melanoma cells. Among other key observations, melanoma cells differ from normal skin melanocytes in EPO-R expression and lack of KIT, suggesting that transformation may be associated with disturbance or deregulation of cytokine receptors. Although distinct subpopulations were also detectable using surface marker antigens, especially an EPO-R+/CD24+ subpopulation, no marker combination was found to be indicative of distinct functional properties or a particular melanoma-initiating potential. Our improving knowledge on phenotypes and phenotypic heterogeneity of melanoma cells may facilitate the detection of these cells and their isolation using mAb directed against various cell surface antigens. The functional and possible pathogenetic role of cytokine receptors and targets expressed in melanoma cells remain to be elucidated.

## Supporting Information

Figure S1 **Gating strategy to detect and characterize primary melanoma cells.** Cells isolated from a primary melanoma lesion (patient #5) were stained with a combination of monoclonal antibodies (Table S1b in File S1). Morphologically viable cells were selected for gating analyses. In a first step, viable cells were gated as CD45−/CD31− cells. In a second step, the CD45−/CD31− cells were examined for expression of CD63 (not shown), CD146, and CD166. Melanoma cells were defined as CD45−/CD31− cells that expressed at least one of the melanoma-related antigens applied, namely CD63, CD146, or CD166.
(TIF)

Figure S2 **Expression of cytokine receptor mRNA species in melanoma cell lines.** Total RNA was isolated from human melanoma cell lines (A375, 607B, Mel-Juso, SK-Mel28) and was subjected to qPCR analysis using primers specific for cytokine receptor genes, other target genes, and a set of control genes (shown in Table S1c in File S1). qPCR was performed as described in the text. Results show the percent of mRNA relative to control (set of house keeping genes = HKGs) and represents the mean±S.D. from three independent experiments. All other mRNA species tested were not expressed in the melanoma cell lines examined: G-CSF-R, ABCG2, KIT, CD20, AC133/CD133, FLT-3/CD135.
(TIF)

Figure S3 **Growth-promoting effect of stem cell factor (SCF) in skin melanocytes.** *A*, Normal epidermal melanocytes (foreskin, Caucasian) were incubated in control medium, recombinant SCF (100 ng/ml), or recombinant erythropoietin, EPO (5 U/ml) at 37°C for 48 hours. Then, ^3^H-thymidine uptake was measured. Results are expressed as percentage of control (no cytokines added = 100%). Data represent the mean±S.D. of 3 independent experiments. *B*, Melanocytes were incubated in control medium, rhSCF (100 ng/ml), rhSCF (100 ng/ml) plus nilotinib (1 µM), or rhEPO (5 U/ml) for 48 hours. Then, ^3^H thymidine uptake was measured. Results represent the mean±S.D. values from triplicates.
(TIF)

File S1 **Table S1 a–f.** Table S1a. Patients' characteristics. Abbreviations: m, male; f, female; NA, information not available; LN, lymph node(s); wt, wild type (of tested region). Table S1b. Combinations of monoclonal antibodies (mAb) applied in four-colour flow cytometry experiments using patient-derived melanoma cells. Abbreviations: FITC, fluorescein isothiocyanate; PE, phycoerythrin; PerCP, peridinin chlorophyll protein; APC, allophycocyanin; EPO-R, erythropoietin receptor; EGF-R, epidermal growth factor receptor; HGF-R, hepatocyte growth factor receptor; IGF-I-R, insulin-like growth factor I receptor; PD-1, programmed death-1; NGF-R, nerve growth factor receptor; HLA, human leukocyte antigens; PECAM-1, platelet/endothelial cell adhesion molecule-1; Siglec-3, sialic acid binding immunoglobulin-like lectin-3; HPCA-1, human progenitor cell antigen-1; LCA, leukocyte common antigen; ICAM-1, intercellular adhesion molecule-1; LAMP-3, lysosomal-associated membrane protein 3; CEACAM-1, carcinoembryonic antigen-related cell adhesion molecule 1; G-CSF-R, granulocyte colony-stimulating factor receptor; CSF-1-R, colony stimulating factor 1 receptor; GM-CSF-R, granulocyte macrophage colony-stimulating factor receptor; SCF, stem cell factor; IL-3Rα, Interleukin 3 receptor, alpha subunit; FLT-3, FMS-like tyrosine kinase 3; Mel-CAM (MCAM), melanoma cell adhesion molecule; ALCAM, activated leukocyte cell adhesion molecule; L1 Antigen/NCAM-1, leukocyte cell adhesion antigen-1/neural cell adhesion molecule-1); MDR-1, multidrug resistance protein-1; VEGF-R2/KDR, vascular endothelial growth factor receptor-2/kinase insert domain receptor; ABCG2, ATP-binding cassette (ABC) transporter subfamily G member 2. Table S1c. Sequences of real-time PCR primers for detection of cytokine receptors- and target genes as well as housekeeping genes. Abbreviations: IGF-I-R, insulin-like growth factor I receptor; EPO-R, erythropoietin receptor; G-CSF-R, granulocyte colony-stimulating factor receptor; ABCG-2, ATP-binding cassette sub-family G member 2; LAMP-3, lysosomal-associated membrane protein 3; VEGF-R, vascular endothelial growth factor receptor; FLT-3, FMS-like tyrosine kinase 3; EEF1A, eukaryotic translation elongation factor 1α1, ACTB, actin beta, RPLP0, ribosomal protein LP0. Table S1d. Expression of cytokine and growth factor receptors in melanoma cell lines, on primary melanoma cells obtained from melanoma patients (melanoma cells cultured in vitro, freshly isolated from patients or xenotransplant-derived) and normal skin melanocytes. Abbreviations: VEGF-R2, vascular endothelial growth factor receptor-2; EPO-R, erythropoietin receptor; IGF-I-R, insulin-like growth factor I receptor; G-CSF-R, granulocyte colony-stimulating factor receptor; GM-CSF-R, granulocyte/macrophage colony-stimulating factor receptor; M-CSF-R, macrophage colony-stimulating factor receptor; SCF, stem cell factor; IL-3RA, interleukin-3 receptor alpha; FLT-3, FMS-like tyrosine kinase 3; n.t., not tested. Table S1e. Expression of adhesion-related molecules in melanoma cell lines, patient-derived melanoma cells and xenotransplanted melanoma cells. Score of reactivity of melanoma cells with antibodies: −, <5%; +/−, 6–20%; +, 21–60%, and ++, 61–100%. Abbreviations: ICAM-1, intercellular adhesion molecule-1; CEACAM-1, carcinoembryonic antigen-related cell adhesion molecule 1. Table S1f. Expression of stem cell-related markers and potential drug targets in melanoma cell lines, patient-derived melanoma cells, and normal epidermal melanocytes. Abbreviations: MDR-1; multidrug resistance gene-1; NGF-R, nerve growth factor receptor; ABCG2, ATP-binding cassette (ABC) transporter subfamily G member 2; Siglec-3, sialic acid binding immunoglobulin-like lectin-3; n.t., not tested.
(DOCX)
