# Supplementary figures and images for: Phenotyping of Human Melanoma Cells Reveals a Unique Composition of Receptor Targets and a Subpopulation Co-Expressing ErbB4, EPO-R and NGF-R
Source: PLoS One. 2014 Jan 29;9(1):e84417. doi: 10.1371/journal.pone.0084417 (PMC3906015; doi:10.1371/journal.pone.0084417)

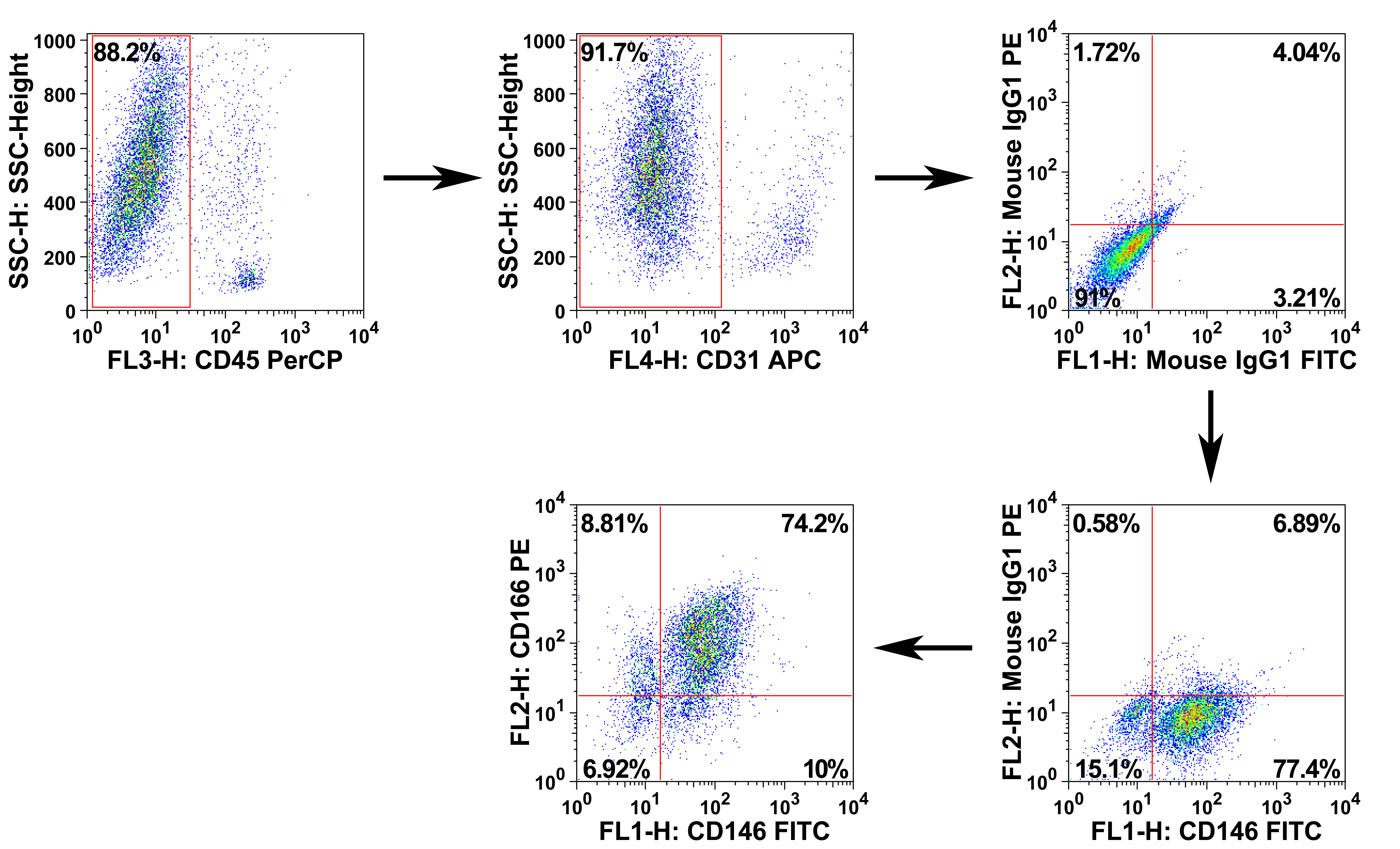

Supplement: Figure S1 — Gating strategy to detect and characterize primary melanoma cells. Cells isolated from a primary melanoma lesion (patient #5) were stained with a combination of monoclonal antibodies (Table S1b in File S1). Morphologically viable cells were selected for gating analyses. In a first step, viable cells were gated as CD45−/CD31− cells. In a second step, the CD45−/CD31− cells were examined for expression of CD63 (not shown), CD146, and CD166. Melanoma cells were defined as CD45−/CD31− cells that expressed at least one of the melanoma-related antigens applied, namely CD63, CD146, or CD166. (TIF) [file pone.0084417.s001.tif]

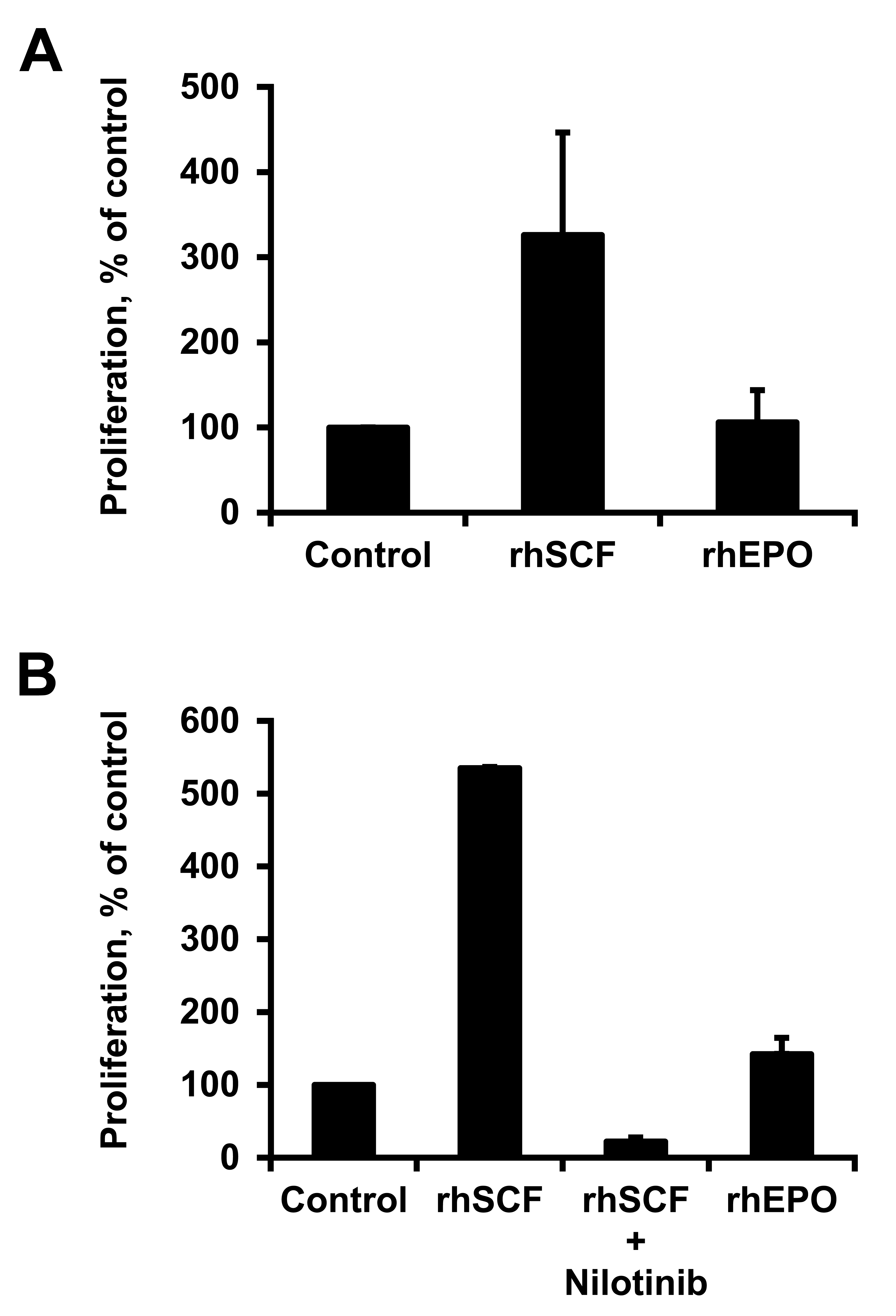

Supplement: Figure S3 — Growth-promoting effect of stem cell factor (SCF) in skin melanocytes. A, Normal epidermal melanocytes (foreskin, Caucasian) were incubated in control medium, recombinant SCF (100 ng/ml), or recombinant erythropoietin, EPO (5 U/ml) at 37°C for 48 hours. Then, 3H-thymidine uptake was measured. Results are expressed as percentage of control (no cytokines added = 100%). Data represent the mean±S.D. of 3 independent experiments. B, Melanocytes were incubated in control medium, rhSCF (100 ng/ml), rhSCF (100 ng/ml) plus nilotinib (1 µM), or rhEPO (5 U/ml) for 48 hours. Then, 3H thymidine uptake was measured. Results represent the mean±S.D. values from triplicates. (TIF) [file pone.0084417.s003.tif]
