# Supplementary material for: Phenotyping of Human Melanoma Cells Reveals a Unique Composition of Receptor Targets and a Subpopulation Co-Expressing ErbB4, EPO-R and NGF-R
Source: PLoS One. 2014 Jan 29;9(1):e84417. doi: 10.1371/journal.pone.0084417 (PMC3906015; doi:10.1371/journal.pone.0084417)
Supplement: File S1 — Table S1 a–f. Table S1a. Patients' characteristics. Abbreviations: m, male; f, female; NA, information not available; LN, lymph node(s); wt, wild type (of tested region). Table S1b. Combinations of monoclonal antibodies (mAb) applied in four-colour flow cytometry experiments using patient-derived melanoma cells. Abbreviations: FITC, fluorescein isothiocyanate; PE, phycoerythrin; PerCP, peridinin chlorophyll protein; APC, allophycocyanin; EPO-R, erythropoietin receptor; EGF-R, epidermal growth factor receptor; HGF-R, hepatocyte growth factor receptor; IGF-I-R, insulin-like growth factor I receptor; PD-1, programmed death-1; NGF-R, nerve growth factor receptor; HLA, human leukocyte antigens; PECAM-1, platelet/endothelial cell adhesion molecule-1; Siglec-3, sialic acid binding immunoglobulin-like lectin-3; HPCA-1, human progenitor cell antigen-1; LCA, leukocyte common antigen; ICAM-1, intercellular adhesion molecule-1; LAMP-3, lysosomal-associated membrane protein 3; CEACAM-1, carcinoembryonic antigen-related cell adhesion molecule 1; G-CSF-R, granulocyte colony-stimulating factor receptor; CSF-1-R, colony stimulating factor 1 receptor; GM-CSF-R, granulocyte macrophage colony-stimulating factor receptor; SCF, stem cell factor; IL-3Rα, Interleukin 3 receptor, alpha subunit; FLT-3, FMS-like tyrosine kinase 3; Mel-CAM (MCAM), melanoma cell adhesion molecule; ALCAM, activated leukocyte cell adhesion molecule; L1 Antigen/NCAM-1, leukocyte cell adhesion antigen-1/neural cell adhesion molecule-1); MDR-1, multidrug resistance protein-1; VEGF-R2/KDR, vascular endothelial growth factor receptor-2/kinase insert domain receptor; ABCG2, ATP-binding cassette (ABC) transporter subfamily G member 2. Table S1c. Sequences of real-time PCR primers for detection of cytokine receptors- and target genes as well as housekeeping genes. Abbreviations: IGF-I-R, insulin-like growth factor I receptor; EPO-R, erythropoietin receptor; G-CSF-R, granulocyte colony-stimulating factor receptor; ABCG-2, [file pone.0084417.s004.docx]

| Patient # | Age | Sex | Tumor localization | Thickness (mm) | Ulceration | Metastases | *B-RAF* status | *N-RAS* status | *KIT* status |
| --- | --- | --- | --- | --- | --- | --- | --- | --- | --- |
| 1 | 59 | m | Multiple sites | NA | NA | skin, pulmonary, hepatic | NA | NA | NA |
| 2 | 58 | f | Back, dorsal | 2.2 | yes | skin, LN, pulmonary | NA | NA | NA |
| 3 | 66 | f | Left thigh | 3 | no | LN, skin, pulmonary | wt | Q61R | wt |
| 4 | 50 | f | Right shank | 2.2 | yes | LN, skin, pulmonary | NA | NA | NA |
| 5 | 66 | f | Left thigh | 3 | no | LN, skin, pulmonary | wt | Q61R | wt |
| 6 | 74 | f | Left feet | 5.2 | yes | skin, LN | NA | NA | NA |
| 7 | 65 | f | Left feet plantar | 5 | yes | LN, pulmonary, cerebral | wt | Q61R | NA |
| 8 | 86 | f | Right thigh | 12 | yes | N.A. | V600E | wt | wt |
| 9 | 56 | m | Right feet | 1.1 | NA | skin, LN | wt | wt | wt |
| 10 | 76 | m | Right feet | 3 | no | skin, LN | wt | wt | D816V |

**Supplementary Table S1a**

*Patients´ characteristics*

Abbreviations: m, male; f, female; NA, information not available; LN, lymph node(s); wt, wild type (of tested region).

**Supplementary Table S1b**

*Combinations of monoclonal antibodies (mAb) applied in four-colour flow cytometry experiments using patient-derived melanoma cells*

| **Tube** | **FITC** | **PE** | **PerCP** | **APC** |
| --- | --- | --- | --- | --- |
| 1 | mIgG1 | mIgG1 | mIgG1 | mIgG1 |
| 2 | CD146 | mIgG1 | CD45 | CD31 |
| 3 | CD146 | mIgG2b | CD45 | CD31 |
| 4 | CD146 | mIgG2a | CD45 | CD31 |
| 5 | CD146 | CD166 | CD45 | CD31 |
| 6 | CD146 | CD63 | CD45 | CD31 |
| 7 | CD146 | CD133 | CD45 | CD31 |
| 8 | CD146 | CD117 | CD45 | CD31 |
| 9 | CD146 | CD20 | CD45 | CD31 |
| 10 | CD146 | CD34 | CD45 | CD31 |
| 11 | CD146 | CD135 | CD45 | CD31 |
| 12 | CD146 | CD243 | CD45 | CD31 |
| 13 | CD146 | ABCG2 | CD45 | CD31 |
| 14 | CD146 | CD44 | CD45 | CD31 |
| 15 | CD146 | CD54 | CD45 | CD31 |
| 16 | CD146 | CD114 | CD45 | CD31 |
| 17 | CD146 | CD115 | CD45 | CD31 |
| 18 | CD146 | CD116 | CD45 | CD31 |
| 19 | CD146 | CD123 | CD45 | CD31 |
| 20 | CD146 | CD105 | CD45 | CD31 |
| 21 | CD146 | CD309 | CD45 | CD31 |
| 22 | CD146 | anti-c-MET/HGFR | CD45 | CD31 |
| 23 | CD146 | anti-IGF-IR | CD45 | CD31 |
| 24 | CD146 | anti-EGFR1/ErbB1 | CD45 | CD31 |
| 25 | CD146 | anti-EGFR2/ErbB2 | CD45 | CD31 |
| 26 | CD146 | anti-EGFR3/ErbB3 | CD45 | CD31 |
| 27 | CD146 | anti-EGFR4/ErbB4 | CD45 | CD31 |
| 28 | CD146 | anti-EPOR | CD45 | CD31 |
| 29 | CD146 | CD66a | CD45 | CD31 |
| 30 | CD146 | CD171 | CD45 | CD31 |
| 31 | CD146 | CD33 | CD45 | CD31 |
| 32 | CD24 | mIgG1 | CD45 | CD31 |
| 33 | mIgG2a | CD146 | CD45 | CD31 |
| 34 | CD24 | CD146 | CD45 | CD31 |
| 35 | mIgG2a | anti-EPOR | CD45 | CD31 |
| 36 | CD24 | mIgG2b | CD45 | CD31 |
| 37 | CD24 | mIgG1 | CD45 | CD31 |
| 38 | CD24 | anti-EPOR | CD45 | CD31 |
| 39 | CD24 | anti-EPOR | CD45 | anti-ErbB4 |
| 40 | CD24 | CD279 | CD45 | anti-ErbB4 |
| 41 | CD24 | CD271 | CD45 | anti-ErbB4 |

Abbreviations: FITC, fluorescein isothiocyanate; PE, phycoerythrin; PerCP, peridinin chlorophyll protein; APC, allophycocyanin; EPO-R, erythropoietin receptor; EGF-R, epidermal growth factor receptor; HGF-R, hepatocyte growth factor receptor; IGF-I-R, insulin-like growth factor I receptor; PD-1, programmed death-1; NGF-R, nerve growth factor receptor; HLA, human leukocyte antigens; PECAM-1, platelet/endothelial cell adhesion molecule-1; Siglec-3, sialic acid binding immunoglobulin-like lectin-3; HPCA-1, human progenitor cell antigen-1; LCA, leukocyte common antigen; ICAM-1, intercellular adhesion molecule-1; LAMP-3, lysosomal-associated membrane protein 3; CEACAM-1, carcinoembryonic antigen-related cell adhesion molecule 1; G-CSF-R, granulocyte colony-stimulating factor receptor; CSF-1-R, colony stimulating factor 1 receptor; GM-CSF-R, granulocyte macrophage colony-stimulating factor receptor; SCF, stem cell factor; IL-3Rα, Interleukin 3 receptor, alpha subunit; FLT-3, FMS-like tyrosine kinase 3; Mel-CAM (MCAM), melanoma cell adhesion molecule; ALCAM, activated leukocyte cell adhesion molecule; L1 Antigen/NCAM-1, leukocyte cell adhesion antigen-1/neural cell adhesion molecule-1); MDR-1, multidrug resistance protein-1; VEGF-R2/KDR, vascular endothelial growth factor receptor-2/kinase insert domain receptor; ABCG2, ATP-binding cassette (ABC) transporter subfamily G member 2.

**Supplementary Table S1c**

*Sequences of real-time PCR primers for detection of cytokine receptors- and*

*target genes as well as housekeeping genes*

| **Gene name** | **GeneID (NCBI)** | **Sequence (5’ – 3’)** |
| --- | --- | --- |
| HER1 (ErbB1) | GeneID:[1956](http://www.ncbi.nlm.nih.gov/entrez/query.fcgi?db=gene&cmd=Retrieve&dopt=full_report&list_uids=1956) | F: GGAGAACTGCCAGAAACTGACC  R: CTGGTTGTGGCAGCAGTCACT |
| HER2 (ErbB2) | GeneID:[2064](http://www.ncbi.nlm.nih.gov/entrez/query.fcgi?db=gene&cmd=Retrieve&dopt=full_report&list_uids=2064) | F: CTGCCGGAGAGCTTTGATG  R: TCAAACACTTGGAGCTGCTCTG |
| HER3 (ErbB3) | GeneID:[2065](http://www.ncbi.nlm.nih.gov/entrez/query.fcgi?db=gene&cmd=Retrieve&dopt=full_report&list_uids=2065) | F: CAACTCTCAGGCAGTGTGTCCT  R: TGTCTGGTATTGGTTCTCAGCATC |
| HER4 (ErbB4) | GeneID:[2066](http://www.ncbi.nlm.nih.gov/entrez/query.fcgi?db=gene&cmd=Retrieve&dopt=full_report&list_uids=2066) | F: GCCATCGAGAATGTGCTGGA  R: AAATTCATGCAGGCAAAGCAG |
| IGF-I-R | GeneID:[3480](http://www.ncbi.nlm.nih.gov/entrez/query.fcgi?db=gene&cmd=Retrieve&dopt=full_report&list_uids=3480) | F: AACACTGGTCATCATGGAACTGA  R: GGAGGTGCTAGGACTGGATTATTC |
| EPO-R | GeneID:[2057](http://www.ncbi.nlm.nih.gov/sites/entrez?db=gene&cmd=Retrieve&dopt=full_report&list_uids=2057) | F: CATCCTGACGCTCTCCCTCA  R: CAGGCCAGATCTTCTGCTTCA |
| G-CSF (CD114) | GeneID:[1441](http://www.ncbi.nlm.nih.gov/sites/entrez?db=gene&cmd=Retrieve&dopt=full_report&list_uids=1441) | F: GCGCGAGCAATAGCAACAA  R: GCTGAAAGGGCCTGATGTTCT |
| ABCG2 (CD338) | GeneID:[9429](http://www.ncbi.nlm.nih.gov/sites/entrez?db=gene&cmd=Retrieve&dopt=full_report&list_uids=9429) | F: GAACGGATTAACAGGGTCATTCA  R: ACGGATAAACTGAGTTCCAACCTT |
| KIT (CD117) | GeneID:[3815](http://www.ncbi.nlm.nih.gov/sites/entrez?db=gene&cmd=Retrieve&dopt=full_report&list_uids=3815) | F: GATTCCCAGAGCCCACAATAGA  R: ATCCACTGGCAGTACAGAAGCA |
| CD44 | GeneID:[960](http://www.ncbi.nlm.nih.gov/sites/entrez?db=gene&cmd=Retrieve&dopt=full_report&list_uids=960) | F: GCATTGCAGTCAACAGTCGAA  R: TCCATTGCCACTGTTGATCACT |
| LAMP-3 (CD63) | GeneID:[967](http://www.ncbi.nlm.nih.gov/sites/entrez?db=gene&cmd=Retrieve&dopt=full_report&list_uids=967) | F: AGGATGCAGGCAGATTTTAAGTG  R: GACTCGGTTCTTCGACATGGA |
| CD24 | GeneID:[100133941](http://www.ncbi.nlm.nih.gov/sites/entrez?db=gene&cmd=Retrieve&dopt=full_report&list_uids=100133941) | F: AGAGACTCAGGCCAAGAAACGT  R: TTTCCTTGCCACATTGGACTT |
| CD20 | GeneID:[931](http://www.ncbi.nlm.nih.gov/sites/entrez?db=gene&cmd=Retrieve&dopt=full_report&list_uids=931) | F: AACTCCAGGAAGTGTTTGGTCAA  R: CATTCCAGAAATGGCAGCAA |
| AC133 (CD133) | GeneID:[8842](http://www.ncbi.nlm.nih.gov/sites/entrez?db=gene&cmd=Retrieve&dopt=full_report&list_uids=8842) | F: CGTCTTCCTCATGGTTGGAGTTG  R: TGCTCGTGTAAGGTTCACAGATCA |
| VEGF-R2 / KDR (CD309) | GeneID:[3791](http://www.ncbi.nlm.nih.gov/sites/entrez?db=gene&cmd=Retrieve&dopt=full_report&list_uids=3791) | F: CTTGGCCTCGGTCATTTATGTC  R: TACACGACTCCATGTTGGTCACTA |
| FLT-3 (CD135) | GeneID:[2322](http://www.ncbi.nlm.nih.gov/sites/entrez?db=gene&cmd=Retrieve&dopt=full_report&list_uids=2322) | F: AGACAAGTCTCCCAACTGCACA  R: GAGTACTGCTCGACACCCACTGT |
| EEF1a1 | [GeneID:1915](http://www.ncbi.nlm.nih.gov/sites/entrez?db=gene&cmd=search&term=1915&RID=859JW7K5013&log$=geneexplicitnucl&blast_rank=4) | F: ATTACAGGGACATCTCAGGCTGAC  R: CATTCTTGGAGATACCAGCTTCAA |
| ACTB | [GeneID:58](http://www.ncbi.nlm.nih.gov/sites/entrez?db=gene&cmd=search&term=58&RID=859TN8M501S&log$=geneexplicitnucl&blast_rank=1) | F: TGGCTCCCGAGGAGCAC  R: TTGAAGGTCTCAAACATGATCTGG |
| RPLP0 | GeneID:[6175](http://www.ncbi.nlm.nih.gov/sites/entrez?db=gene&cmd=Retrieve&dopt=full_report&list_uids=6175) | F: CATCTACAACCCTGAAGTGCTTGA  R: CATTGCGGACACCCTCCAG |

Abbreviations: IGF-I-R, insulin-like growth factor I receptor; EPO-R, erythropoietin receptor; G-CSF-R, granulocyte colony-stimulating factor receptor; ABCG-2, ATP-binding cassette sub-family G member 2; LAMP-3, lysosomal-associated membrane protein 3; VEGF-R, vascular endothelial growth factor receptor; FLT-3, FMS-like tyrosine kinase 3; EEF1A, eukaryotic translation elongation factor 1α1, ACTB, actin beta, RPLP0, ribosomal protein LP0.

| **Supplementary Table S1d** | | |  |  |  |  |  |  |  |  | |  | |  | |  | |  | |  | |
| --- | --- | --- | --- | --- | --- | --- | --- | --- | --- | --- | --- | --- | --- | --- | --- | --- | --- | --- | --- | --- | --- |
|  |  |  |  |  |  |  |  |  |  |  | |  | |  | |  | |  | |  | |
| *Expression of cytokine and growth factor receptors in melanoma cell lines, on primary melanoma cells obtained from melanoma* | | | | | | | | | | | | | | | | | | | |  | |
| *patients (melanoma cells cultured in vitro, freshly isolated from patients or xenotransplant-derived) and normal skin melanocytes* | | | | | | | | | | | | | | | | | | | | | |
|  |  |  |  |  |  |  |  |  |  |  | |  | |  | |  | |  | |  | |
|  | VEGFR2 | ErbB1 | ErbB2 | ErbB3 | ErbB4 | IGF-I-R | Endoglin | KIT | EPO-R | c-MET | | G-CSFR | | GM-CSFR | | M-CSFR | | IL-3RA | | FLT-3 | |
| **CELL LINES** |  |  |  |  |  |  |  |  |  |  | |  | |  | |  | |  | |  | |
| A375 | 04 | 0 | 0 | 93 | 02 | 93 | 95 | 0 | 0 | 93 | | 0 | | 0 | | 0 | | 0 | | 03 | |
| 607B | 01 | 0 | 0 | 81 | 03 | 35 | 96 | 0 | 0 | 94 | | 0 | | 0 | | 0 | | 0 | | 01 | |
| Mel-Juso | 03 | 0 | 0 | 95 | 02 | 79 | 95 | 0 | 0 | 54 | | 0 | | 0 | | 0 | | 0 | | 04 | |
| SK-Mel28 | 02 | 0 | 0 | 91 | 03 | 45 | 96 | 0 | 0 | 13 | | 0 | | 0 | | 0 | | 0 | | 0 | |
|  |  |  |  |  |  |  |  |  |  |  | |  | |  | |  | |  | |  | |
| **PRIMARY MELANOMA CELLS CULTURED *IN VITRO* OR XENOTRANSPLANTED (XT) MELANOMA CELLS** | | | | | | | | | | | | | | | | | | | |  | |
| #1 Xt | n.t. | n.t. | n.t. | 64 | n.t. | 36 | 63 | 0 | n.t. | n.t. | | n.t. | | n.t. | | n.t | | n.t. | | n.t. | |
| #2 Xt | n.t. | 0 | 0 | 17 | 0 | 0 | n.t. | n.t. | n.t. | n.t. | | n.t. | | n.t. | | n.t. | | n.t. | | n.t. | |
| #3 Xt | n.t. | n.t. | n.t. | 12 | n.t. | 08 | n.t. | 0 | 10 | n.t. | | n.t. | | n.t. | | n.t. | | n.t. | | n.t. | |
| #4 Xt | n.t. | n.t. | n.t. | 69 | n.t. | 16 | n.t. | 0 | 19 | n.t. | | n.t. | | n.t. | | n.t. | | n.t. | | n.t. | |
| #5 | n.t. | 0 | 0 | n.t. | n.t. | 0 | 50 | n.t. | 7 | 0 | | n.t. | | n.t. | | n.t. | | n.t. | | n.t. | |
| #6 | n.t. | 0 | 0 | 02 | n.t. | 0 | 32 | 0 | n.t. | n.t. | | n.t. | | n.t. | | n.t. | | n.t. | | n.t. | |
| #7 | n.t. | 0 | 0 | 35 | n.t. | 03 | 43 | 0 | 16 | n.t. | | n.t. | | n.t. | | n.t. | | n.t. | | n.t. | |
| #8 | n.t. | 0 | 0 | 03 | n.t. | 0 | 32 | 0 | n.t. | n.t. | | n.t. | | n.t. | | n.t. | | n.t. | | n.t. | |
| #9 | n.t. | 0 | 0 | 09 | n.t. | 01 | 55 | 0 | n.t. | n.t. | | n.t. | | n.t. | | n.t. | | n.t. | | n.t. | |
|  |  |  |  |  |  |  |  |  |  |  | |  | |  | |  | |  | |  | |
| **PATIENT-DERIVED MELANOMA** **CELLS, FRESHLY ISOLATED** | | | | | | | |  |  |  | |  | |  | |  | |  | |  | |
| #1 | n.t. | 0 | n.t. | n.t. | n.t. | n.t. | n.t. | 0 | n.t. | n.t. | | n.t. | | n.t. | | n.t. | | n.t. | | n.t. | |
| #2 | n.t. | 0 | n.t. | n.t. | n.t. | n.t. | 01 | 0 | n.t. | n.t. | | n.t. | | n.t. | | n.t. | | n.t. | | n.t. | |
| #3 | n.t. | 0 | 07 | 31 | n.t. | 31 | 12 | 0 | 08 | 0 | | n.t. | | n.t. | | n.t. | | n.t. | | n.t. | |
| #4 | n.t. | n.t. | n.t. | n.t. | n.t. | n.t. | n.t. | n.t. | 04 | n.t. | | n.t. | | n.t. | | n.t. | | n.t. | | n.t. | |
| #5 | 0 | n.t. | n.t. | 57 | n.t. | n.t. | n.t. | 0 | 06 | n.t. | | n.t. | | n.t. | | n.t. | | n.t. | | 0 | |
| #6 | 0 | 0 | 0 | 52 | n.t. | 0 | 08 | 03 | 40 | n.t. | | n.t. | | n.t. | | n.t. | | n.t. | | 0 | |
| #7 | 04 | 0 | 0 | 81 | 50 | 06 | n.t. | 0 | 17 | n.t. | | n.t. | | n.t. | | n.t. | | n.t. | | 0 | |
| #8 | 02 | 0 | 0 | 73 | 36 | 21 | 02 | 0 | 36 | n.t. | | n.t. | | n.t. | | n.t. | | n.t. | | 0 | |
| #9 | 0 | 0 | 0 | 55 | 02 | 03 | 26 | 0 | 04 | 0 | | 0 | | n.t. | | n.t. | | n.t. | | 0 | |
| #10 | 02 | 0 | 0 | 71 | 24 | 12 | 09 | 04 | 27 | 0 | | n.t. | | n.t. | | n.t. | | n.t. | | n.t. | |
|  |  |  |  |  |  |  |  |  |  | |  |  |  | |  | |  | |  | |  |
|  |  |  |  |  |  |  |  |  |  |  | |  | |  | |  | |  | |  | |
|  | VEGFR2 | ErbB1 | ErbB2 | ErbB3 | ErbB4 | IGF-I-R | Endoglin | KIT | EPO-R | c-MET | | G-CSFR | | GM-CSFR | | M-CSFR | | IL-3RA | | FLT-3 | |
|  | | | | | | | | | | | | | | | | | |  | |  | |
| **PATIENT-DERIVED SUBFRACTIONS** **OF XENOTRANSPLANTED (XT) MELANOMA CELLS** | | | | | | | | | | | | | | | | | |  | |  | |
| #7 Xt | 0 | 0 | 05 | 82 | 28 | 48 | 06 | 15 | 29 | 0 | | 0 | | 0 | | 0 | | 0 | | 0 | |
| (EPOR+/-) |  |  |  |  |  |  |  |  |  |  | |  | |  | |  | |  | |  | |
|  |  |  |  |  |  |  |  |  |  |  | |  | |  | |  | |  | |  | |
| #6 Xt | 0 | 0 | 13 | 86 | 12 | 20 | 24 | 08 | 17 | 0 | | n.t. | | 0 | | 0 | | 0 | | 0 | |
| (EPOR+/-) |  |  |  |  |  |  |  |  |  |  | |  | |  | |  | |  | |  | |
|  |  |  |  |  |  |  |  |  |  |  | |  | |  | |  | |  | |  | |
| #6 Xt 2nd | 0 | 0 | n.t. | n.t. | 38 | n.t. | n.t. | n.t. | 42 | 0 | | n.t. | | n.t. | | n.t. | | n.t. | | n.t. | |
| (EPOR+) |  |  |  |  |  |  |  |  |  |  | |  | |  | |  | |  | |  | |
|  |  |  |  |  |  |  |  |  |  |  | |  | |  | |  | |  | |  | |
| **NORMAL HUMAN EPIDERMAL MELANOCYTES (NHEMs)** | | | | | | | | | | | |  | |  | |  | |  | |  | |
| #1 | n.t. | n.t. | 0 | 95 | 0 | 14 | 94 | 95 | 0 | n.t. | | n.t. | | n.t. | | n.t. | | n.t. | | n.t. | |
| #2 | n.t. | n.t. | 0 | 95 | 0 | n.t. | n.t. | 98 | 06 | n.t. | | n.t. | | n.t. | | n.t. | | n.t. | | n.t. | |
| #3 | n.t. | n.t. | n.t. | n.t. | n.t. | n.t. | n.t. | 96 | 0 | n.t. | | n.t. | | n.t. | | n.t. | | n.t. | | n.t. | |
| #4 | n.t. | n.t. | n.t. | n.t. | n.t. | n.t. | n.t. | 98 | n.t. | n.t. | | n.t. | | n.t. | | n.t. | | n.t. | | n.t. | |
| #5 | n.t. | n.t. | n.t. | n.t. | n.t. | n.t. | n.t. | 96 | 01 | n.t. | | n.t. | | n.t. | | n.t. | | n.t. | | n.t. | |

Abbreviations: VEGF-R2, vascular endothelial growth factor receptor-2; EPO-R, erythropoietin receptor; IGF-I-R, insulin-like growth factor I receptor; G-CSF-R, granulocyte colony-stimulating factor receptor; GM-CSF-R, granulocyte/macrophage colony-stimulating factor receptor; M-CSF-R, macrophage colony-stimulating factor receptor; SCF, stem cell factor; IL-3RA, interleukin-3 receptor alpha; FLT-3, FMS-like tyrosine kinase 3; n.t., not tested.

| **Supplementary Table S1e** | | | |
| --- | --- | --- | --- |
|  |  |  |  |
| *Expression of adhesion-related molecules in melanoma cell lines, patient-derived* | | | |
| *melanoma cells and xenotransplanted melanoma cells* | | | |
|  |  |  |  |
|  | ICAM-1/CD54 | CEACAM-1/CD66a | L1/CD171 |
| **MELANOMA CELL LINES** | |  |  |
| A375 | 95 | 0 | 94 |
| 607B | 95 | 85 | 93 |
| Mel-Juso | 95 | 0 | 73 |
| SK-Mel28 | 58 | 95 | 93 |
|  |  |  |  |
| **PATIENT-DERIVED MELANOMA CELLS** | | |  |
| #5 | 72 | 48 | 0 |
| #6 | 86 | 37 | 0 |
| #7 | 97 | 01 | 0 |
| #8 | 74 | 71 | 0 |
| #9 | 63 | 22 | 0 |
| #10 | 47 | 53 | 0 |
|  |  |  |  |
| **PATIENT-DERIVED XENOTRANSPLANTED** | | |  |
| #7 Xt | 93 | 17 | 0 |
| (EPOR+/-) |  |  |  |
|  |  |  |  |
| #6 Xt | 94 | 66 | 0 |
| (EPOR+/-) |  |  |  |

Score of reactivity of melanoma cells with antibodies: -, < 5%; +/-, 6-20%; +, 21-60%,

and ++, 61-100%.

Abbreviations: ICAM-1, intercellular adhesion molecule-1;

CEACAM-1, carcinoembryonic antigen-related cell adhesion molecule 1.

| **Supplementary Table S1f** | | | | |  |  |  |  |  |
| --- | --- | --- | --- | --- | --- | --- | --- | --- | --- |
|  |  |  |  |  |  |  |  |  |  |
| *Expression of stem cell-related markers and potential drug targets in melanoma cell lines,* | | | | | | | | | |
| *patient-derived melanoma cells, and normal epidermal melanocytes* | | | | | | | | |  |
|  |  |  |  |  |  |  |  |  |  |
|  | AC133 CD133 | CD20 | MDR-1 CD243 | ABCG2 CD338 | Pgp-1 CD44 | CD24 | Siglec-3 CD33 | NGF-R CD271 | PD1 CD279 |
| **CELL LINES** | | |  |  |  |  |  |  |  |
| A375 | 0 | 0 | 0 | 0 | 100 | 10 | 25 | 55 | 0 |
| 607B | 0 | 0 | 0 | 0 | 100 | 07 | 60 | 75 | 0 |
| Mel-Juso | 0 | 0 | 0 | 0 | 100 | 11 | 0 | 18 | 0 |
| SK-Mel28 | 0 | 0 | 0 | 0 | 100 | 06 | 0 | 29 | 0 |
|  |  |  |  |  |  |  |  |  |  |
| **PATIENT CELLS CULTURED OR XENOTRANSPLANTED (Xt)** | | | | | | | | |  |
| #1 Xt | 07 | n.t. | n.t. | n.t. | 52 | n.t. | n.t. | n.t. | n.t. |
| #2 Xt | 0 | n.t. | n.t. | 01 | n.t. | n.t. | n.t. | n.t. | n.t. |
| #3 Xt | 0 | 03 | n.t. | 10 | n.t. | n.t. | 07 | n.t. | n.t. |
| #4 Xt | 04 | 02 | n.t. | 0 | n.t. | n.t. | 31 | n.t. | n.t. |
| #5 | 0 | n.t. | n.t. | n.t. | 60 | n.t. | 01 | n.t. | n.t. |
| #6 | 0 | n.t. | n.t. | n.t. | n.t. | n.t. | n.t. | n.t. | n.t. |
| #7 | 0 | 0 | 0 | 03 | 52 | n.t. | 26 | n.t. | n.t. |
| #8 | 0 | n.t. | n.t. | n.t. | 27 | n.t. | n.t. | n.t. | n.t. |
| #9 | 0 | n.t. | n.t. | 0 | 75 | n.t. | n.t. | n.t. | n.t. |
|  |  |  |  |  |  |  |  |  |  |
| **PATIENT-DERIVED FRESHLY ISOLATED MELANOMA CELLS** | | | | | | | | |  |
| #1 | 0 | n.t. | n.t. | n.t. | 01 | n.t. | n.t. | n.t. | n.t. |
| #2 | 0 | n.t. | n.t. | n.t. | 02 | n.t. | n.t. | n.t. | n.t. |
| #3 | 01 | 0 | 01 | 05 | 15 | n.t. | 07 | n.t. | n.t. |
| #4 | 0 | n.t. | n.t. | 01 | n.t. | n.t. | 02 | n.t. | n.t |
| #5 | 0 | 0 | 0 | 01 | n.t. | 10 | 01 | n.t. | n.t. |
| #6 | 0 | 0 | 0 | 0 | 95 | 48 | 0 | n.t. | n.t. |
| #7 | 0 | 0 | 0 | 0 | 95 | 15 | 0 | n.t. | n.t. |
| #8 | 0 | 0 | 0 | 0 | 78 | 36 | 03 | 22 | 23 |
| #9 | 37 | 0 | 0 | 0 | 70 | 04 | 0 | n.t. | n.t. |
| #10 | 0 | n.t. | n.t. | 05 | 70 | 25 | 0 | n.t. | n.t. |
|  |  |  |  |  |  |  |  |  |  |
| **XENOTRANSPLANTED (Xt) MELANOMA CELL SUBSETS** | | | | | | | |  |  |
| #7 Xt | 0 | 0 | 0 | 0 | 98 | 29 | 0 | 01 | 13 |
| (EPOR+/-) |  |  |  |  |  |  |  |  |  |
|  |  |  |  |  |  |  |  |  |  |
| #6 Xt | 0 | 0 | 0 | 0 | 98 | 11 | 0 | n.t. | n.t. |
| (EPOR+/-) |  |  |  |  |  |  |  |  |  |
|  |  |  |  |  |  |  |  |  |  |
| #6 Xt 2nd | n.t. | n.t. | n.t. | n.t. | n.t. | 38 | n.t. | 02 | 16 |
| (EPOR+) |  |  |  |  |  |  |  |  |  |
|  |  |  |  |  |  |  |  |  |  |
| **NORMAL EPIDERMAL MELANOCYTES** | | | | | | |  |  |  |
| #1 | 05 |  |  | 0 | 98 | 0 | 0 | 19 | 0 |
| #2 | n.t. |  |  | n.t. | n.t. | n.t. | n.t. | 26 | 0 |

Abbreviations: MDR-1; multidrug resistance gene-1; NGF-R, nerve growth factor receptor; ABCG2, ATP-binding cassette (ABC) transporter subfamily G member 2; Siglec-3, sialic acid binding immunoglobulin-like lectin-3; n.t., not tested.
